# An association between elevated telomerase reverse transcriptase expression and the immune tolerance disruption of dendritic cells

**DOI:** 10.1186/s12964-024-01650-6

**Published:** 2024-05-23

**Authors:** Xuejie Xu, Lihua Mo, Yun Liao, Kaitlyn Song Zhang, Hanqing Zhang, Le Liu, Yu Liu, Aifa Tang, Pingchang Yang, Xiaoyu Liu

**Affiliations:** 1 Institute of Allergy & Immunology of Shenzhen University and State Key Laboratory of Respiratory Diseases Allergy Division, Shenzhen University, Xueyuan Blvd, Shenzhen, A7-511. 1066, 518500 China; 2 Department of General Medicine Practice, Third Affiliated Hospital of Shenzhen University, Shenzhen, China; 3 Shenzhen Clinical College, Guangzhou University of Chinese Traditional Medicine & Pharmaceutics, Guangzhou, China; 4 The Country Day School, King City, ON Canada

**Keywords:** Allergy, Nose, Mucosa, Dendritic cell, Telomerase

## Abstract

**Background:**

To elucidate the mechanism of dysfunction of tolerogenic dendritic cells (DCs) is of significance. Telomerase involves the regulation of the cell fate and activities. The objective of this study is to investigate the role of telomerase reverse transcriptase (TERT) in regulating the tolerogenic feature of DCs.

**Methods:**

The telomerase was assessed in DCs, which were collected from patients with allergic rhinitis (AR), healthy control (HC) subjects, and mice. RNAs were extracted from DCs, and analyzed by RNA sequencing (RNAseq), real-time quantitative RT-PCR, and Western blotting.

**Results:**

The results showed that expression of *TERT* was higher in peripheral DCs of AR patients. The expression of *IL10* in DCs was negatively correlated with the levels of *TERT* expression. Importantly, the levels of *TERT* mRNA in DCs were associated with the AR response in patients with AR. Endoplasmic reticulum (ER) stress promoted the expression of *Tert* in DCs. Sensitization with the ovalbumin-aluminum hydroxide protocol increased the expression of *Tert* in DCs by exacerbating ER stress. TERT interacting with c-Maf (the transcription factor of IL-10) inducing protein (CMIP) in DCs resulted in CMIP ubiquitination and degradation, and thus, suppressed the production of IL-10. Inhibition of *Tert* in DCs mitigated experimental AR.

**Conclusions:**

Elevated amounts of TERT were detected in DCs of patients with AR. The tolerogenic feature of DCs was impacted by TERT. Inhibited TERT attenuated experimental AR.

**Supplementary Information:**

The online version contains supplementary material available at 10.1186/s12964-024-01650-6.

## Introduction

Dendritic cells (DCs) are a fraction of immune cells in the body. It is known that DCs are the first line of immune response. DCs process and present antigens to T cells. Together with propter cytokines and processed antigens, DCs initiate certain immune responses. DC1s [X-C Motif Chemokine Receptor 1 (XCR1)^+^CD11c^+^; also called myeloid DCs] produce IL-12, and initiate T helper (Th)1 response. DC2s (also called lymphoid DCs; CD103^+^CD172a^+^) initiate Th2 response [1–3]. Tolerogenic DCs (TolDCs) are a subtype of DCs, CD141^+^ [1–3]. Semi-maturation is the feature of TolDCs based on the low expression MHC II, CD80, CD86. One of the features of TolDCs is the production of immune regulatory molecules, such as interleukin (IL)-10, which can in turn induce the development of TolDCs [3, 4]. In terms of tolerogenic, TolDCs can induce regulatory T cells; such as type 1 regulatory T cells (Tr1 cells). TolDC-derived IL-10 plays a critical role in the induction of Tr1 cells [5]. The deficiency or insufficiency of IL-10 in DCs has been linked to the pathogenesis of many immune diseases, such as allergic diseases and autoimmune diseases [6]. To date, the pathogenesis of dysfunction of TolDCs has not been elucidated yet. Factors regulating the amounts of IL-10 in DCs remain to be further elucidated.

Published data indicate that telomerase is involved in the pathogenesis of immune inflammation [7]. The essential role of telomerase is to maintain the homeostasis of the telomere [8]. Besides, telomerases are also involved in a variety of cell activities. For example, telomerase reverse transcriptase (TERT), a catalytic enzyme, can interact with protein kinase B (PKB/AKT) and rapamycin (mTOR) [9]. The phosphatidylinositol 3-kinase (PI3K)-Akt-mTOR signal pathway plays an essential role in different immune regulatory activities, including allergic inflammation [10]. Yet, the mechanism behind TERT regulation has not been fully understood.

The homeostasis of immune cells can be disturbed by endoplasmic reticulum (ER) stress, which can affect their functions, as previously reported [11]. ER stress occurs in the cell in response to various stimuli. Such a condition induces the cell to synthesize a large number of proteins to restore the homeostasis in the cell [12]. Meanwhile, the unfolded or misfolded proteins may aggregate in the ER. If the stimuli are too strong or last for a too long time, unfolded protein response (UPR) may occur. Some of the ER stress associated molecules may be over expressed, which may disturb the immune homeostasis and induce inflammation in the body [13]. Yet, whether UPR alters telomerase activity and further influences DC’s immune tolerant features is unknown. In this study, we observed an elevated TERT expression in peripheral DCs of patients with allergic rhinitis (AR). TERT caused CMIP (c-Maf inducing protein), the transcription factor of *IL10*, to ubiquitinate and degrade, resulting in a decrease in DCs’ tolerogenic capacity.

## Results

### The expression of telomerase is negatively associated with the expression of IL-10 in DCs

**Fig. 1:**
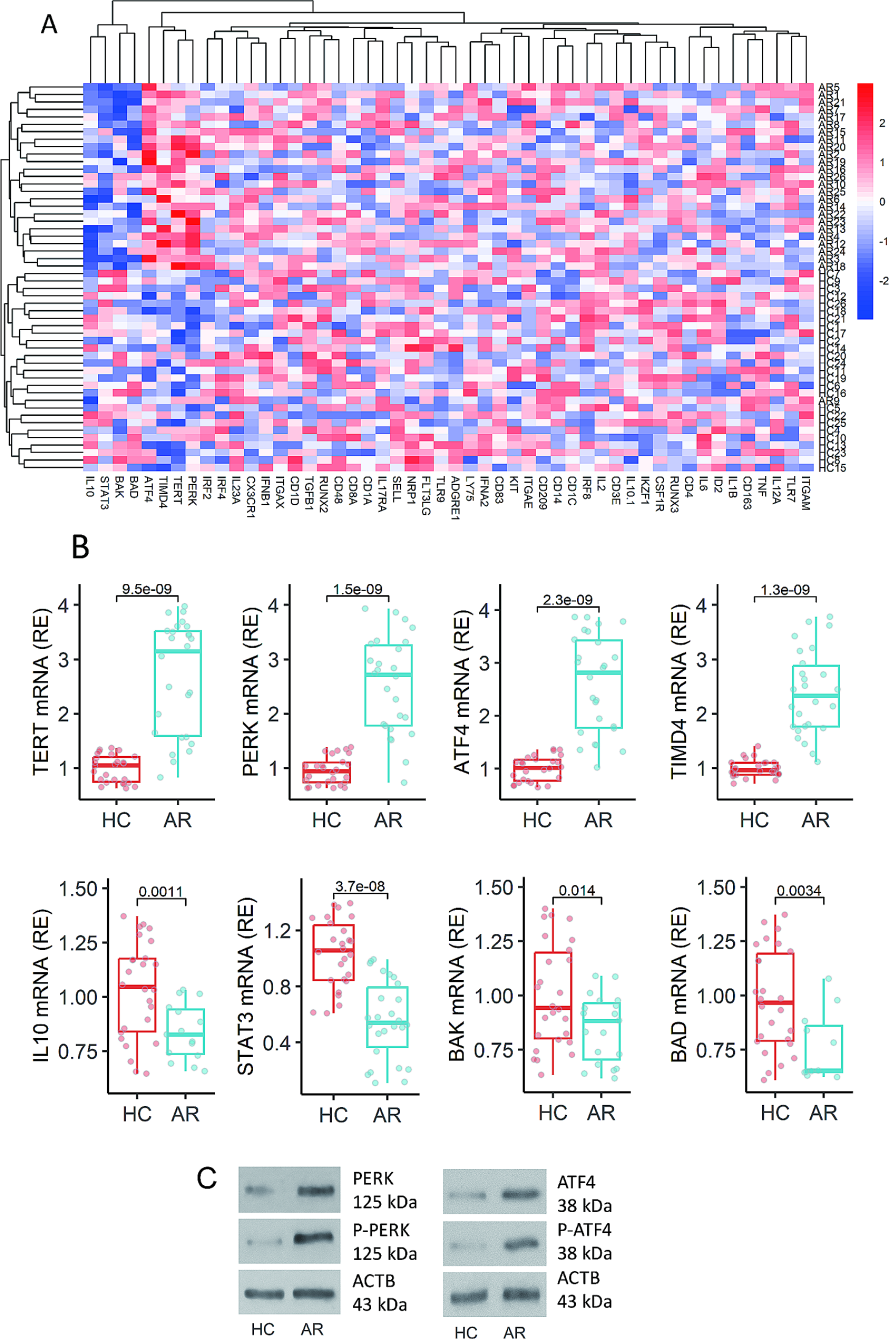
Peripheral DCs of AR patients show higher expression of *TERT*, higher ER stress status, and lower *IL10* expression. **A**-**B**, peripheral DCs were obtained from 26 AR patients and 26 HC subjects, and analyzed by RNAseq. **A**, a heatmap shows the expression of top 50 DEGs in individual samples (the data are clustered according to the adjusted *p* values). **B**, RNA samples of DC were analyzed by conventional RT-qPCR (each sample was tested in triplicate). Boxplots show the expression of up and down regulated DEGs. **C**, protein extracts of DCs were analyzed by Western blotting. Immunoblots show the amounts of total and phosphorylated ATF4 and PERK in DCs. Each dot in boxplots presents one sample. Statistics: Mann Whitney test. *p* values are provided in figures where appropriate. Data of immunoblots are from one experiment that represent 3 independent experiments with pooled samples per group. Abbreviations: HC: Healthy control. AR: Allergic rhinitis. DC: Dendritic cell. RNAseq: RNA sequencing assay. DEG: Differentially expressed gene

Blood samples from 26 AR patients and 26 HC subjects were collected. DCs were isolated from the samples by flow cytometry (FCM) cell sorting (Fig. S1 in supplemental materials). RNA samples were extracted from DCs, and analyzed by RNA sequencing (RNAseq). The results showed *TERT* (variant 2), *PERK, EIF2A, TIMD4* in the up regulated 38 differentially expressed genes (DEGs), whereas *IL10, STAT3, BAK* and *BAD* were included in the 23 down regulated DEGs (Fig. 1A-B). The results of RNAseq were verified by conventional RT-qPCR (Fig. 1C). Western blotting assay showed elevated amounts of total and phosphorylated PERK and EIF2A protein in AR DCs (Fig. 1D). The lower expression of *IL10* in AR tolerogenic DCs (TolDCs of AR subjects, the IL-10^+^ DCs) was verified in separate experiments (Fig. S2A-C). The expression of *TGFB* and *PDL1* was comparable between conventional DCs (cDCs) and AR TolDCs or HC TolDCs (Fig. S2D-E). TolDCs from both AR and HC subjects showed lower expression of *CD80* and *CD86* (Fig. S2F-G). The data were analyzed by Spearman’s correlation coefficient assay. A positive correlation between the expression of *TERT* and those ER stress associated molecule expression was detected in DCs, while the expression of *IL10* and *STAT3* was negatively correlated with those ER stress associated molecules in DCs (Fig. S3). The results implicate that both ER stress and telomerase TERT may be involved in regulating the expression of *IL10* in DCs.

### The expression of telomerase in DCs is associated with the AR response

**Fig. 2:**
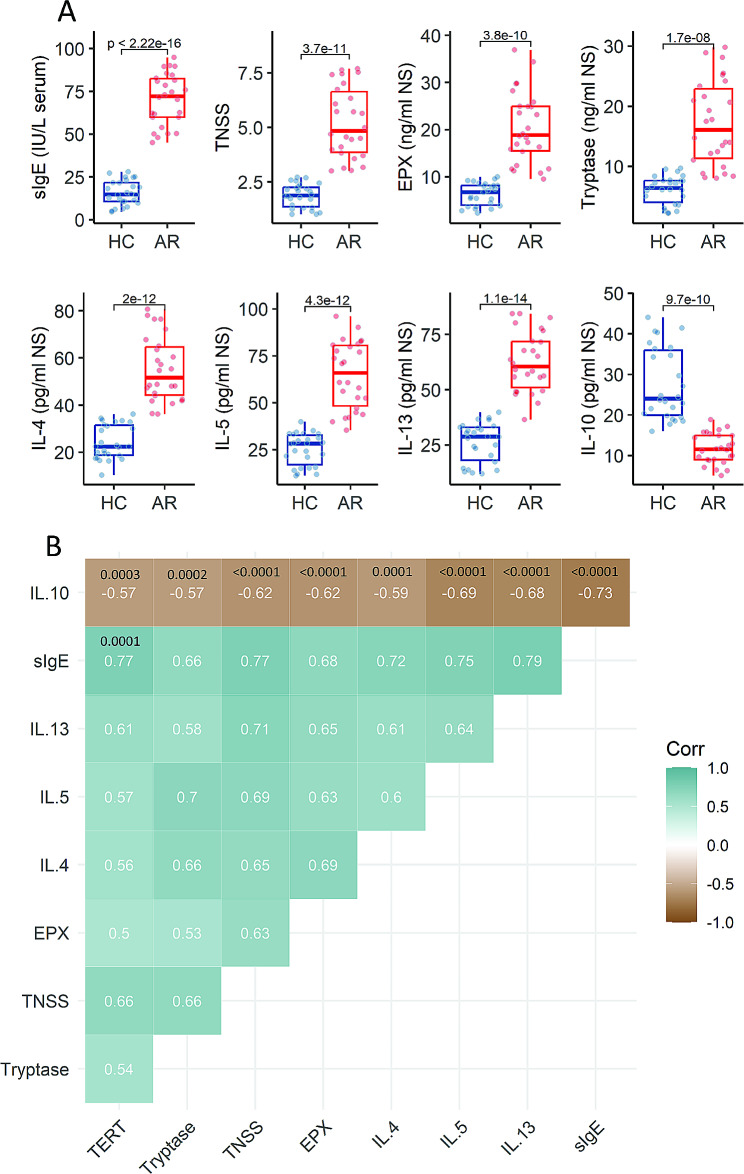
The expression of *TERT* in DCs is correlated with the AR response. **A**, boxplots show the AR response. The data of boxplots are presented as median (IQR) from 26 samples per group. Each dot in boxplots presents one sample (each sample was tested in triplicate). **B**, a heatmap show the correlation between the expression of *TERT* in peripheral DCs and the AR response. The coefficients are presented in each square (in white). Statistics: Mann Whitney test (**A**) and Spearman correlation coefficient test (**B**). *P* values (in black) are provided in figures where appropriate. Abbreviations: HC: Healthy control. AR: Allergic rhinitis. DC: Dendritic cell

The expression of IL-10 is the mainstay of the tolerogenic DCs (TolDCs) [14]. The reduction of IL-10 production in TolDCs significantly affects the tolerogenic function of DCs, which is one of the features in the pathogenesis of AR [15]. Prompted by the results of Fig. 1, we deduced that the expression of TERT in DCs might be associated with the pathogenesis of AR. We then assessed the association between telomerase in DCs and the AR response. The AR response include high amounts of specific IgE in the serum, high amounts of allergy relative mediators (eosinophil peroxidase and tryptase), and Th2 cytokines (IL-4, IL-5, IL-13) in nasal secretions, and high total nasal symptom scores (TNSS) (Fig. 2A). The data showed a positive correlation between the TERT mRNA expression and the AR response. The amounts of IL-10 in the NS were negatively correlated with the expression of *TERT* in DCs (Fig. 2B). The results show that the expression of telomerase in peripheral DCs is associated with the pathogenesis of AR.

### ER stress promotes the expression of *Tert* in DCs

**Fig. 3:**
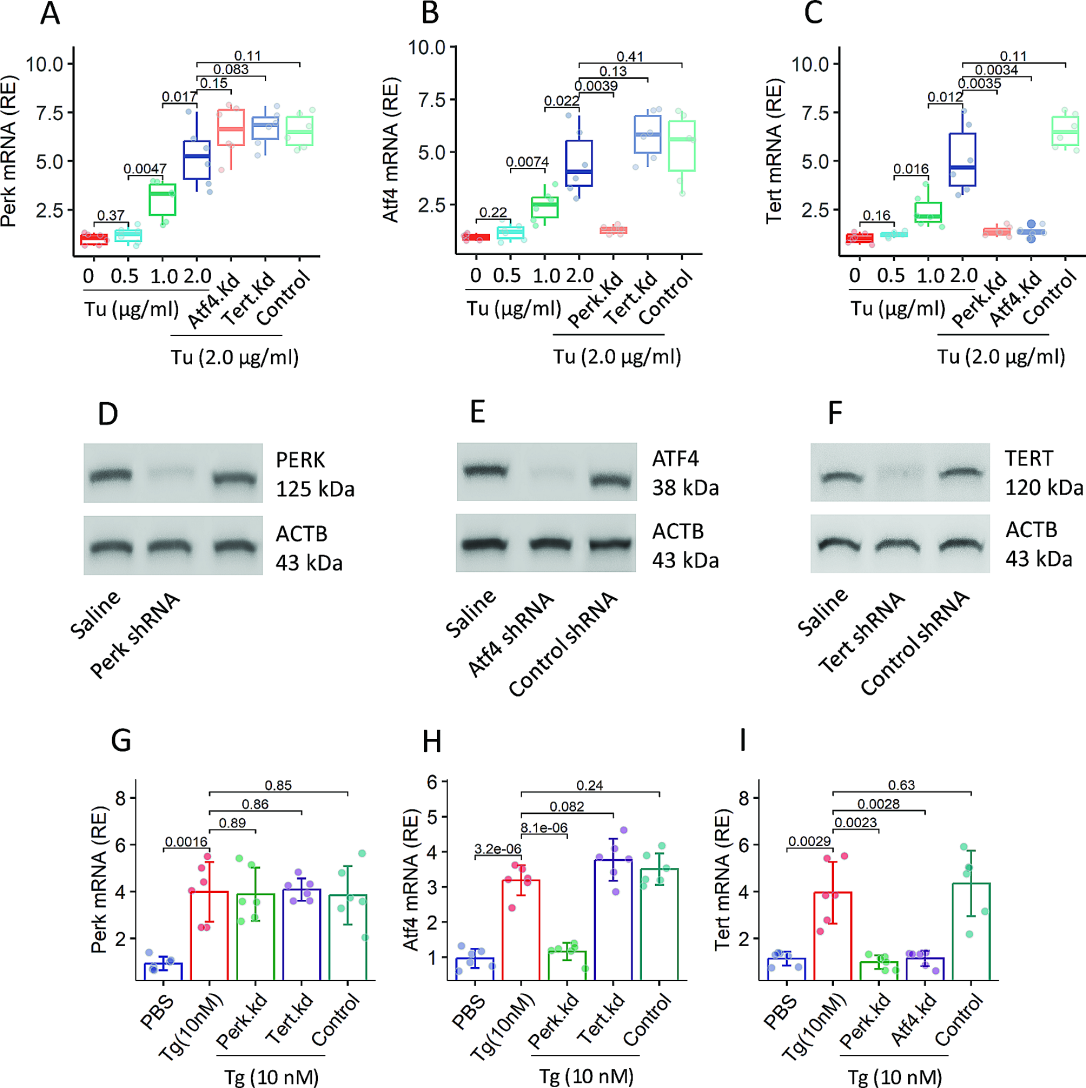
ER stress modulates the expression of *Tert* in DCs. Naïve DCs were exposed to Tu in culture for 48 h. **A**-**C**, RNA extracts of DCs were analyzed by RT-qPCR. Boxplots show median (IQR) of mRNA levels of *Perk* (**A**), *Atf4* (**B**) and *Tert* (**C**), respectively. **D**-**F**, immunoblots show the results of RNAi of *Perk* (**D**), *Atf4* (**E**), and *Tert* (**F**), respectively. **G**-**I**, DCs were treated with the conditions denoted on the X axis of barplots. Bars show mean ± SD of mRNA amounts of indicated molecules in DCs. Statistics: ANOVA followed by the Bonferroni test. *p* values are presented in figures where appropriate. Each dot in boxplots presents one sample (each sample was tested in triplicate). The data of immunoblots are from one experiment that represent 3 independent experiments with pooled samples per group. Abbreviations: ER: Endoplasmic reticulum. DC: Dendritic cell. Tu: Tunicamycin. Kd: Knockdown. Tg: Thapsigargin

We examined the effects of ER stress on the regulation of Tert expression in DCs. Naïve DCs were isolated from the mouse spleen, and cultured in the presence of tunicamycin (Tu, in short, an agonist of ER stress [16]) for 48 h. Tu exposure resulted in a dose-dependent increase in *Tert*, *Perk*, and *Atf4* expression in DCs (Fig. 3A-C). Knockdown of the expression of *Atf4* abolished the Tu-induced expression of *Tert* in DCs, while knockdown of the expression of *Tert* did not alter the Tu-induced expression of *Perk* or *Atf4* (Fig. 3A-F). To corroborate the results, DCs were treated with an alternative ER stress agonist, thapsigargin, with the same experimental setting of Fig. 3A-C. Similar results were obtained (Fig. 3G-I). The results indicate that ER stress can induce the expression of TERT in DCs through the PERK-ATF4 signaling pathway.

### Sensitization promotes the expression of TERT by exacerbating ER stress in DCs

**Fig. 4:**
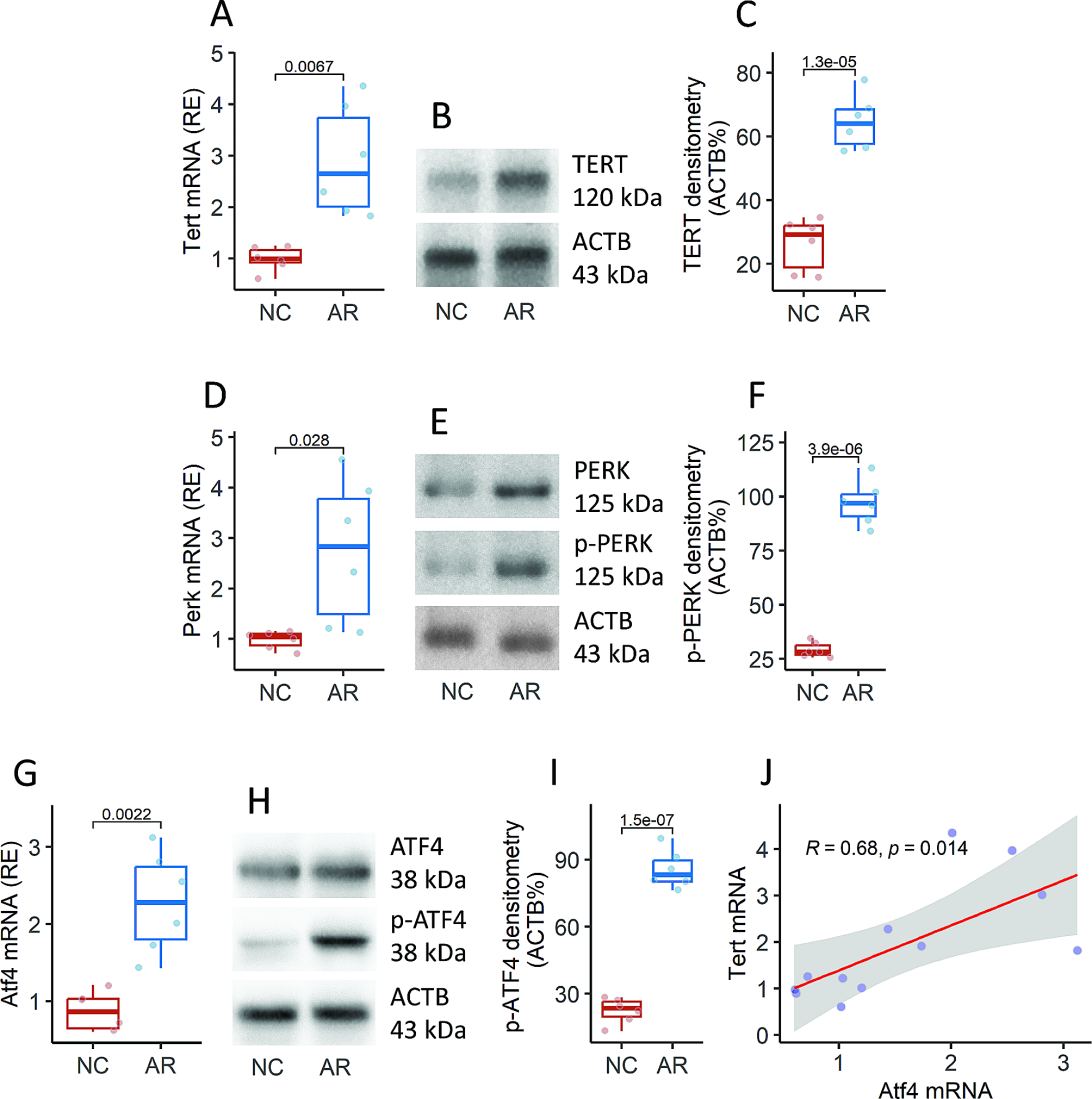
Sensitization induces the expression of TERT and activation of PERK and ATF4 in DCs of the airway tissues. Mice were treated with the OVA-alum protocol to establish an AR mouse model. DCs were isolated from the airway tissues. RNA and protein extracts of DC were prepared and analyzed by RT-qPCR and Western blotting. **A**, **D** and **G**, the mRNA levels of *Tert*, *Perk* and *Atf4* in DCs. B, E and H, the densitometry of TERT, p-PERK and p-ATF4 immunoblots. **C**, **F** and **I**, the densitometry results of the immunoblots. Data of immunoblots are from one experiment that represent three independent experiments with pooled samples per group. **J**, positive correlation between the mRNA levels of *Atf4* and *Tert* in DCs. The data in boxplots are presented as median (IQR) from 6 mice per group. Each dot in boxplots presents one sample (each sample was tested in triplicate). Statistics: Student *t*-test (boxplots) and Pearson correlation coefficient test (J). *P* values are provided in figures where appropriate. The experiments were repeated three times. Abbreviations: NC: Naïve control. AR: Allergic rhinitis. OVA: Ovalbumin. DC: Dendritic cell. p-PERK: the “p” stands for “phosphorylated”

Mice were sensitized using the ovalbumin (OVA)-aluminum hydroxide (alum) protocol to establish an AR mouse model following our established protocol [17] (Fig. S4). The mice showed the AR-like response, including nasal itch, sneezing, increase in the amounts of eosinophil peroxidase (EPX) and mouse mast cell protease-1 (Mcpt1), increase in IL-4, IL-5, and IL-13 in the nasal lavage fluids (NLF). The immune suppressive cytokine, IL-10, in NLF was reduced. The amounts of specific IgE in the serum were also increased (Fig. S5). DCs were isolated from the airway tissues and subjected to the analysis with RT-qPCR and Western blotting. The results showed that DCs of the sensitized mice (AR mice) expressed higher amounts of *Tert*, *Perk* and *Atf4* than in the naive control (NC) mice. A positive correlation was detected between the mRNA levels of *Tert* and *Atf4* in DCs (Fig. 4). The findings indicate that TERT expression in DCs may be influenced by sensitization by exacerbating ER stress.

### TERT interacts with CMIP in DCs to interfere with the expression of IL-10

**Fig. 5:**
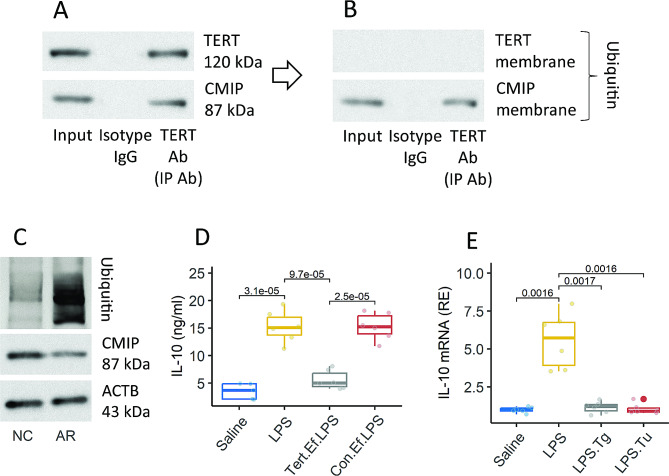
TERT induces CMIP degradation and suppresses IL-10 production by DCs. **A**-**C**, DCs were isolated from AMCs of mice with or without AR. **A**, immunoblots show a complex of TERT and CMIP in protein extracts of DC. **B**, ubiquitin was colocalized with CMIP. **C**, ubiquitination of CMIP. **D**, naïve DCs were treated as denoted on the X axis for 24 h. Boxplots show median (IQR) of IL-10 amounts in culture supernatant. **E**, DCs were cultured in the presence of LPS (10 µg/ml) or with or without Tg or Tu overnight. DCs were then analyzed by RT-qPCR. Boxplots show median (IQR) of *Il10* mRNA. Each dot in boxplots presents one sample (each sample was tested in triplicate). Statistics: ANOVA followed by the Bonferroni test. *p* values are presented in figures where appropriate. The experiments were repeated three times. Abbreviations: NC: Normal control. AR: Allergic rhinitis. AMC: Airway mononuclear cell. Tert.Ef: Enforced expression of *Tert*. Con.Ef: DCs were transfected with control plasmids used as control. Tg: Thapsigargin. Tu: Tunicamycin

DCs were isolated from the airway tissues of mice with AR. Proteins were extracted from DCs and analyzed by immunoprecipitation (IP) with an anti-TERT Ab as a bait. The IP products were analyzed by Western blotting. A complex of TERT and CMIP was detected (Fig. 5A). The membrane was further treated with the peel-re-staining procedures. Ubiquitin was colocalized with the CMIP, but not TERT (Fig. 5B). Ubiquitination staining was used to analyze the protein extracts. Stronger ubiquitination was detected in CMIP, which was reversed with the amounts of CMIP in DCs (Fig.). 5 C). The results suggest that TERT physically interacts with CMIP to promote the ubiquitination and degradation of CMIP. Since CMIP is the transcription factor of IL-10, the results implicate that TERT may suppress the expression of IL-10 in DCs. To test this, naïve DCs were transfected with TERT expression plasmids (Fig. S6), and exposed to LPS (Sigma Aldrich; the inducer of IL-10 in DCs) for two days. The TERT plasmid transfection resulted in the expression of TERT in DCs, which effectively suppressed the expression of IL-10 in DCs induced by LPS (Fig. 5D). Alternatively, the presence ER stress agonists also interfered with the induction of IL-10 in DCs (Fig. 5E). The results indicate that TERT can suppress the expression of IL-10 in DCs by inducing the degradation of CMIP. In other words, sensitization may exacerbate the ER stress, promote Tert expression, and disrupt DC’s immune tolerance function, and consequently, contribute to the development of airway allergy.

### Inhibition of *Tert* in DCs mitigates development of AR

**Fig. 6:**
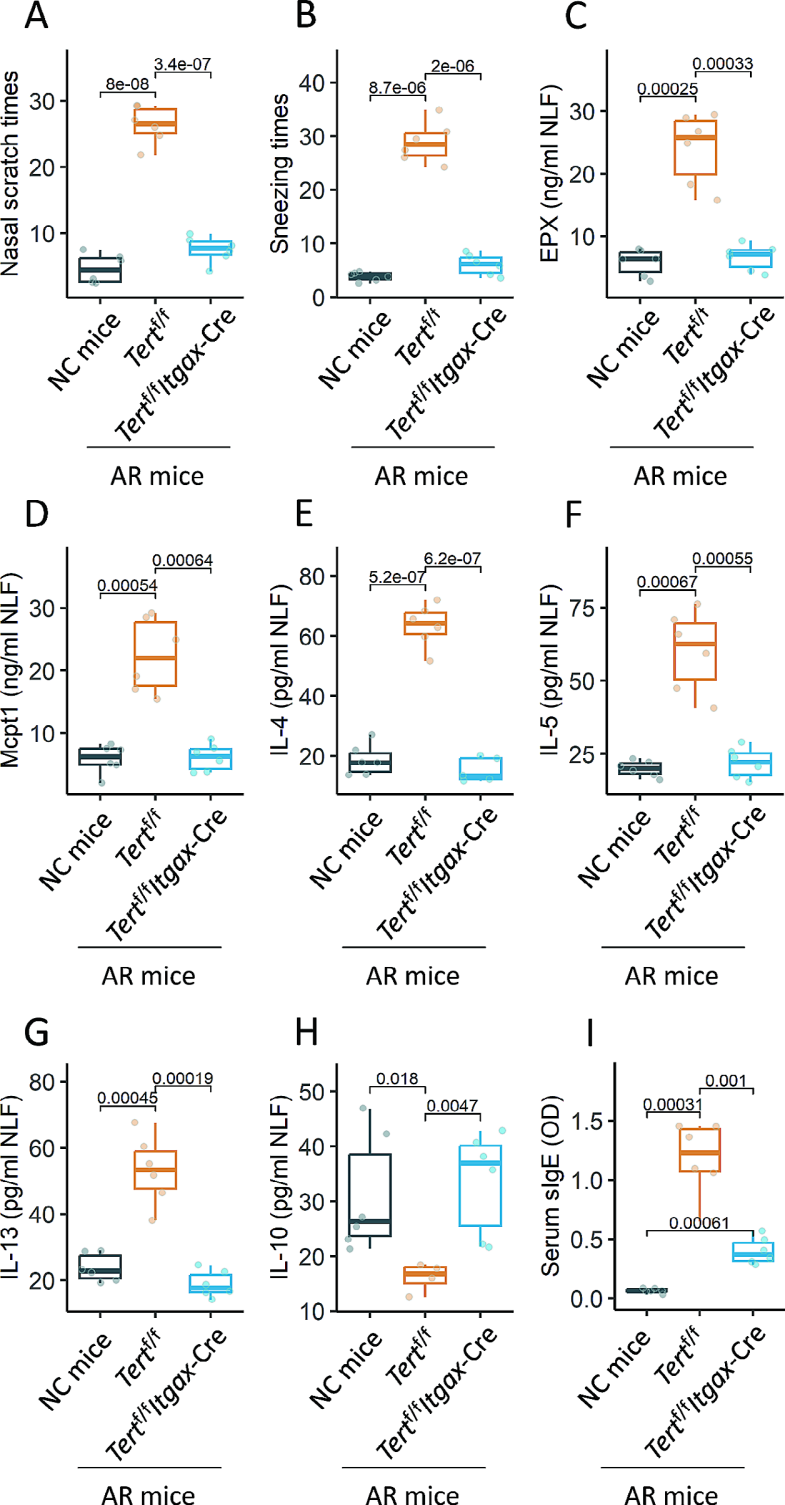
Inhibition of *Tert* in DCs attenuates experimental AR. *Tert*^f/f^ mice and *Tert*^f/f^*Itgax*-Cre mice were treated with the OVA-alum protocol to establish an AR mouse model. Boxplots show the AR response, including AR symptoms (nasal itch and sneezing, **A** and **B**), increase in allergic mediators (EPX and Mcpt1, **C** and **D**), increase in Th2 cytokines (IL-4, IL-5, and IL-13, E-G), and decrease in IL-10 (H) in NLF, and increase in sIgE in the serum (I). Each group consists of 6 mice. The data of boxplots are presented as median (IQR). Each dot in boxplots presents one sample (each sample was tested in triplicate). Statistics: Student *t*-test. P values are presented in figures where appropriate. The experiments were repeated three times. Abbreviations: NC: Naïve control. AR: Allergic rhinitis. DC: Dendritic cell. NLF: Nasal lavage fluid

To further explore the role of *Tert* expression in DC’s immune regulatory capacity, a mouse strain with conditional ablation of the *Tert* gene in DCs was generated in-house. The gene ablation did not affect the frequency of immune cells and DC functions in the airway tissues of the mice (Fig. S7). An AR mouse model was established using the OVA-alum protocol (Fig. S4). The gene ablation system of *Tert*^f/f^*Itgax*-Cre mice was activated by feeding mice with tamoxifen prior to the sensitization. The sensitized *Tert*^f/f^ mice showed the AR responses, including AR symptoms (nasal itch and sneezing), increase allergic mediators (eosinophil peroxidase and mouse mast cell protease-1) and Th2 cytokines (IL-4, IL-5 and IL-13) in NLF (nasal lavage fluids), and the increase in serum sIgE. As expected, the AR response was significantly mitigated in sensitized *Tert*^f/f^*Itgax*-Cre mice (Fig. 6). In addition, we also found that mice treated with a neutralizing anti-IL-10 Ab through nasal instillations for one week resulted in Th2 bias in the airways (Fig. S8). The results indicate that *Tert* expression is crucial in the pathogenesis of AR.

### *Tert* suppression promotes immunotherapy of AR

**Fig. 7:**
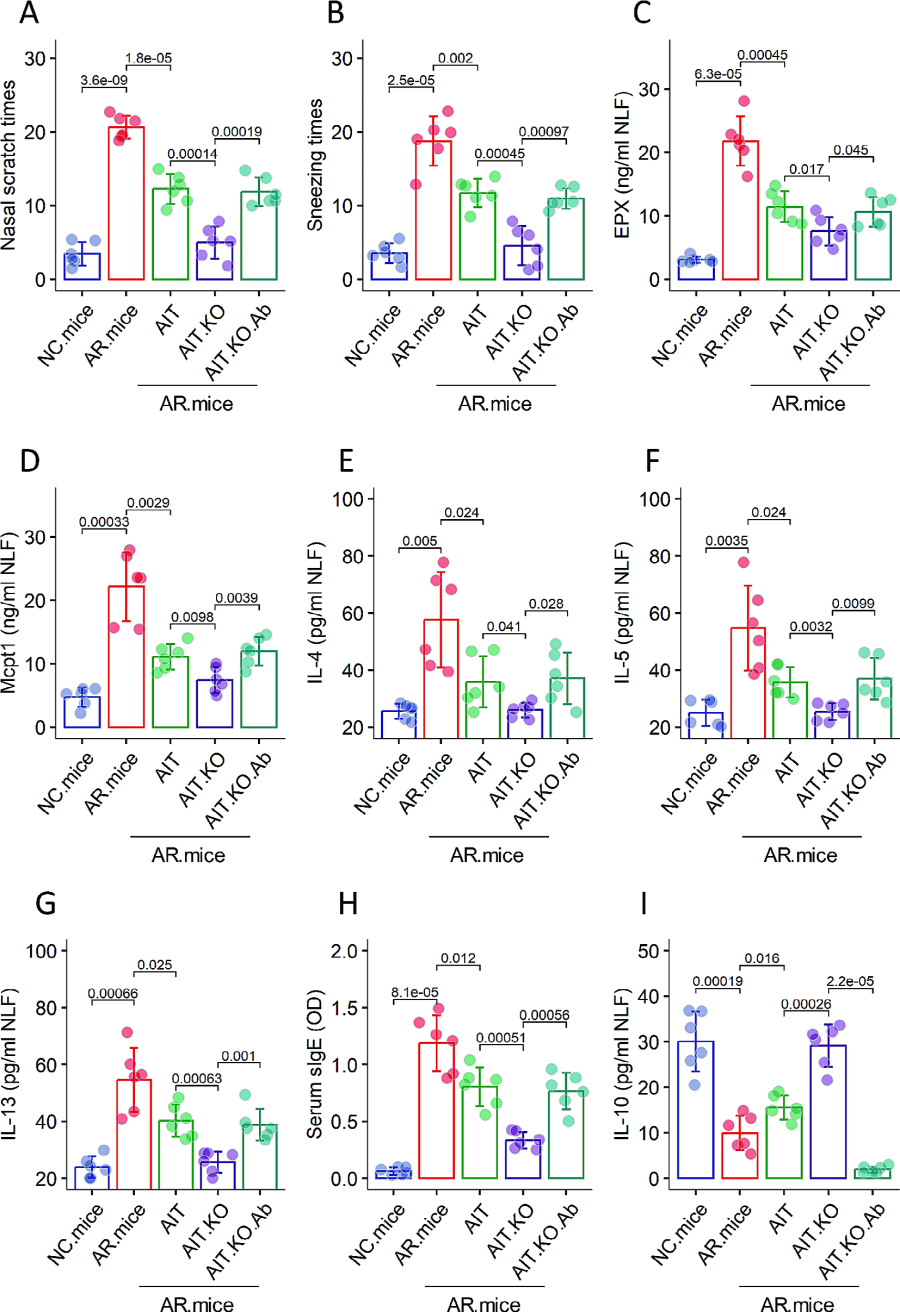
Suppression of *Tert* in DCs promotes therapeutic effects of AIT on AR. An AR mouse model was established with the protocol illustrated in Fig. S4. The *Tert*^f/f^ mice were employed for the groups of NC.mice, AR.mice, and AR.mice.AIT, respectively. The *Tert*^f/f^*Itgax*-Cre mice were employed for the AIT.KO group. Bar graphs show mean ± SD of the amounts of indicated molecules in NLF and serum. Each dot in bars presents one sample (each sample was tested in triplicate). Statistics: ANOVA + Bonferroni test. p values are presented in figures where appropriate. The experiments were repeated three times. Abbreviations: NC: Naïve control. AR: Allergic rhinitis. AIT: Allergen specific immunotherapy. DC: Dendritic cell. KO: *Tert*^f/f^*Itgax*-Cre mice were fed with tamoxifen after the completion of sensitization. Ab: IL-10 neutralizing Ab (JES5-2A5)

We then tested the effects of suppression of Tert in DCs on promoting the therapeutic effects of allergen specific immunotherapy (AIT) on experimental AR. An AR mouse model was established following the protocol in Fig. S4 with *Tert*^f/f^ mice and *Tert*^f/f^*Itgax*-Cre mice. The gene ablation system of *Tert*^f/f^*Itgax*-Cre mice was activated by feeding mice with tamoxifen after the completion of the sensitization. The AR mice were treated with AIT with or without the ablation of the *Tert* gene in DCs. The results showed that the AIT had therapeutic effects on the AR response, which were markedly promoted by the ablation of the *Tert* gene in DCs, which was abolished by the administration of an IL-10 neutralizing Ab (Fig. 7). The results suggest that inhibition of the *Tert* expression in DCs has the therapeutic effects on AR.

## Discussion

The present study revealed that the expression of *TERT*, which encodes for the catalytic subunit of telomerase, in DCs was elevated in subjects with AR. The AR responses were positively correlated with the expression of *TERT* in DCs. CMIP is an enhancer of the IL-10 [18] expression in DCs. The interaction between TERT and CMIP in DCs resulted in CMIP degradation. This resulted in a suppression of the expression of IL-10 by DCs. Consequently, the immune tolerogenic feature in DCs was impaired. The inhibition of the expression of *Tert* in DCs effectively prevented the development of AR as well as promoted the therapeutic effects of AIT on AR in mice.

The data show that the expression of *TERT* in DCs was higher in AR patients as compared with HC subjects. Maintaining the homeostasis of the telomeres is the primary function of telomerase. However, cumulative reports indicate that besides the basic role of maintaining and repairing telomeres, telomerases are also linked to the pathogenesis of many inflammatory disorders. Our results implicate that the increase in TERT in DCs may link to the pathogenesis of AR. Previous reports indicate that the over expression of TERT is associated with the pathogenesis of diabetes, rheumatoid arthritis, and systemic lupus erythematosus [7]. Current data show that the amounts of TERT in DCs are in parallel to the AR response parameters in patients. DCs are the important immune cell fraction playing a critical role in the earliest immune response in the development of AR [15]. The data suggests that the increase in TERT expression in DCs is a factor that is affecting the homeostasis in DCs.

The data show that the amounts of CMIP are less in DCs of AR subjects compared to those in control subjects. CMIP is an important factor in the production of c-Maf, the transcription factor of IL-10, inducing the expression of IL-10 [18]. The pathogenesis of immune diseases is associated with the reduction of c-Maf, as indicated by previous reports. For example, Hooper et al. found that prostaglandin E could suppress c-Maf and the expression of IL-10 to inhibit the type 1 regulatory T cell differentiation [19]. It is well known that exposure to lipopolysaccharide (LPS) can induce the expression of *IL10* [20], c-Maf mediates the effects of LPS on inducing the IL-10 gene transcription [18]. Our data also show that the decrease in the amounts of CMIP in DCs is in parallel to the decrease in the expression of IL-10. Such a phenomenon is consistent with previous reports, in which Wang et al. found that CMIP played a critical role in the expression of *IL10* in B cells. The circadian locomotor output cycles kaput (CLOCK) interacted with CMIP to cause degradation, which reduced IL10 expression in B cells [21].

To find the causative factors in the reduction of the IL-10 expression in DCs is of significance. Our data show that the expression of *TERT* is negatively correlated with the amounts of CMIP as well as the expression of *Il10* in DCs. However, the expression of *CMIP* was not altered in DCs isolated from the blood samples collected from AR patients as well as DCs isolated from the airway tissues of AR mice. The fact suggests that the decrease in the amounts of CMIP in DCs of AR subjects occurs in post transcription. Indeed, a complex of CMIP and TERT was found in DCs collected from AR subjects. In particular, ubiquitin and CMIP were found to be colocalized in the complex. As ubiquitination is one of the mechanisms of protein degradation [22], the phenomenon indicates that TERT promotes the degradation of CMIP in DCs of subjects with AR. Since CMIP is the critical factor of the expression of IL-10 [18, 21], the data indicate that the over expression of TERT is an important factor in the suppression of the IL-10 expression in DCs.

IL-10 is the primary factor in the tolerogenic feature of immune regulatory cells [23, 24]. DCs interact with a variety of immune cells to regulate the activities of other immune cells [25]. The reduction of the IL-10 expression suggests a dysfunctional state of immune regulatory cells [23, 26]. A novel point of the present study is that we found an overexpression of *Tert* in DCs of subjects with AR, which could disrupt the tolerogenic feature of TolDCs. Thus, it is reasonable to deduce that to suppress the expression of *Tert* can restore the tolerogenic feature of TolDCs. The data verified our hypothesis. By conditional ablation of *Tert* in DCs, the core of tolerogenic feature, IL-10, was maintained in airway DCs of mice treated with the OVA-alum protocol. The AR responses were thus mitigated.

Additionally, while the study demonstrates that *Terf*^f/f^*Itgax*-Cre mice do not exhibit defects in the frequency of T cells, B cells, and DCs, it is worth exploring whether DCs in the conditional KO demonstrate any other potential defects beyond the TERT-CMIP-IL-10 axis with such a strong reduction in AR. On the other hand, it is known that TolDCs induce type 1 regulatory T cells (Tr1 cells) [5]. Whether TERT involves in regulating the development of Tr1 cells is an interesting topic to be further investigated. Previous reports indicate that TERT is involved in cancer, inflammation and immune disorders [7, 27, 28]. Current study focuses on the role of TERT in the pathogenesis of AR. TERT may also be involved in other immune responses that need to be explored in the future.

In summary, the study found that DCs from AR subjects displayed high levels of TERT. The expression of IL-10 in DCs was suppressed by TERT. To inhibit the TERT expression efficiently suppressed experimental AR by maintaining the tolerogenic feature of TolDCs.

### Limitation of the study

A portion of data was generated from murine model experiments. Dendritic cells collected from human airways, such as those from bronchoalveolar lavage fluids, may provide further information about the role of telomerase in the regulation of dendritic cell properties.

## Materials and methods

### Reagents

shRNA kits of *Tert*, *Perk* and *Atf4*, antibodies (Abs) of TERT (Clone#: C-12, Cat#: sc-377,511), ATF4 (B-3, sc-390,063), CD19 (B-1, sc-390,244, fluorochrome: AF488), MHC II (7–1 H): sc-13,556, AF546), CD11c (G-3, sc-398,725, AF594), CD14 (H-4, sc-515,785; 61D3, sc-52,457, AF648), CD16 (YFC 120.5, sc-58,962, AF700), CD163 (ED2, sc-58,965, AF790), CD141 (D-3, sc-13,164, AF488), CD103 (OX62, sc-53,085, AF546), CD172a (C-7, sc-376,884, AF594) and F4/80 (D-11, sc-365,340, AF648) were purchased from Santa Cruz Biotech (Santa Cruz, CA). Abs of PERK (ab229912), p-PERK (phospho T982; ab192591), p-ATF4 (phospho S245; ab28830) were purchased from abcam (Cambridge, MA). CMIP Ab was purchased from MyBioSource (MBS9603486; San Diego, CA). Ab of XCR1-PE, ELISA kits of EPX, tryptase, Mcpt1, OVA-specific IgE, IL-4, IL-5, IL-10, and IL-13 were purchased from Dakewe BioMart (Shenzhen, China). IL-10 neutralizing Ab (JES5-2A5) was bought from eBioscience (San Diego, CA). Reagents and materials for RT-qPCR, Western blotting and IP were purchased from Invitrogen (Carlsbad, CA).

### Ethics statement

The study protocol was approved by the human ethics committee and the animal ethics committee at our institution. A written informed consent was obtained from each human subject. All animal experiments were conducted in accordance to the ARRIVE guidelines.

### Human subjects

Patients with perennial allergic rhinitis (AR) were enrolled into this study. The diagnosis of AR was carried out by our experienced physicians based on the routine procedures established in our department, which can be found elsewhere [29, 30]. Briefly, the inclusive criterion of AR was that patients had perennial AR for more than two years, positive skin prick test results, and positive serum specific IgE. Subjects with any of the following conditions were excluded, which were severe organ diseases, cancers, autoimmune diseases, and those under treatment with immune suppressive agents for any reason. In addition, healthy control (HC) subjects were also enrolled, who had no allergic disease history, negative skin prick test results, and negative serum sIgE. The human ethics committee at our institution approved the human experimental protocol (approve #2022-0036). A written informed consent was obtained from each human subject. The demographic data are presented in Table 1.

**Table 1:** Demographic data of human subjects

Items	AR patients	HC subjects
Number	26	26
Age (years)	32.46 ± 4.88	30.89 ± 3.78
Male (%)	13 (50)	13 (50)
Female (%)	13 (50)	13 (50)
FEV1 (% predicted)	99.64 (97.6, 102.1)	100.68 (96.8,102.2)
Serum IgE (IU/ml)	336.8 ± 48.2	20.2 ± 8.9
Serum sIgE (positive, %)	26 (100)	0
Co-suffer allergy		
Allergic asthma	2 (7.7%)	0
Allergic dermatitis	2 (7.7%)	0
Food allergy	4 (15.4%)	0
Using corticosteroids#	26 (100%)	0
Blood neutrophil (10^9^/L)	5.31 (4.68, 6.15)	5.28 (4.66, 5.83)
Blood eosinophil (10^9^/L)	0.46 (0.21, 0.68)	0.16 (0.12, 0.28)
SPT results		
Mite mix	26 (100%)	0
Timothy grass	4 (15.4%)	0
Bermuda grass	3 (11.53%)	0
Pine	2 (7.7%)	0
Mold mix	2 (7.7%)	0
Poplar	3 (11.53%)	0
Rye	1 (3.8%)	0
Mugwort	1 (3.8%)	0
Animal dander	2 (7.7%)	0

The data are presented as means ± SD or median (IQR).

FEV1: Forced expiratory volume in 1 s;

Specific IgE (sIgE) > 0.35 IU/ml was considered as positive

#, patients used corticosteroid spray to control AR attacks

### Collection of nasal secretions (NS)

A piece of degreasing cotton (about 1 g) was gently placed in the middle nasal meatus. It was taken out 5 min later. NS was squeezed out from the cotton, and analyzed in other experiments.

### Total nasal symptom score (TNSS)

TNSS was recorded from each human subject following reported method [31]. TNSS includes three parts: 1, nasal obstruction (scored 0, 1, 2, 3), nasal itching/sneezing (scored 0, 1, 2, 3), secretion/nasal discharge (scored 0, 1, 2, 3). Human subjects were asked to record TNSS daily for one week prior to the enrollment.

### Real-time quantitative RT-qPCR (RT-qPCR)

RNAs were extracted from cells harvested from relevant experiments. The first strand of cDNA was synthesized with the RNA samples using a reverse transcription kit following the manufacturer’s instructions. In the presence of relevant primers (Table S1 in supplemental materials), the samples were amplified in a qPCR device (Bio Rad CFX96) using the SYBR Green Master Mix. The results were calculated with the 2^-∆∆Ct^ method with the reference gene β-actin, and presented as relative expression (RE).

### Preparation of mouse airway mononuclear cells (AMCs)

The lungs were excised right after the sacrifice, cut into small pieces, incubated with collagenase IV (0.5 mg/ml) for 20 min at 37 °C with mild agitation. Single cells were filtered through a cell strainer (70 μm first, then 40 μm). AMCs were isolated from the single cells by the Percoll gradient density centrifugation.

### Establishment of an AR mouse model

Following the established procedures [17], mice were treated with the ovalbumin (OVA)-aluminum hydroxide (alum) protocol as illustrated in Fig. S4 in the supplemental materials. The animal experimental protocols were approved by the Animal Ethics Committee of Shenzhen University (Approve#2022-008).

### Assessment of the AR responses in mice

The AR responses in mice include the AR-like clinical symptoms (nasal itch and sneezing), the amounts of allergic mediators [eosinophil peroxidase (EPX) and mouse mast cell protease-1 (Mcpt1)], and Th2 cytokines (IL-4, IL-5, and IL-13) in the nasal lavage fluid (NLF), and increase in the amounts of specific IgE (sIgE). The AR response was evaluated following the established procedures of our laboratory that also can be found in our previous reports [17].

### Collection of NLF

The trachea was opened in the neck right after the sacrifice. A syringe needle was inserted into the trachea towards the nasal direction. A saline solution (1 ml per mouse) was injected into the upper respiratory tract and recovered from the nostrils.

### Colocalization of CMIP and ubiquitin

The PVDF membrane of the IP experiment for detecting the CMIP-TERT complex was treated with peel-re-staining procedures and stained with ubiquitin Ab following the Western blotting procedures.

### RNA interference (RNAi)

DCs were transfected with the shRNA kits (Santa Cruz Biotech) of *Perk*, or *Atf4*, or *Tert*, or control reagents following the manufacturer’s instruction. The effects of RNAi were checked in the cells by Western blotting 2 days after the transfection.

### Over expression of TERT in DCs

Naïve DCs were transfected with *Tert*-expression plasmids (labeled with His, provided by the San Gong Biotech, Shanghai, China) or control plasmids following the manufacturer’s instruction. The recombinant TERT in DCs were checked by Western blotting two days later.

### Preparation of RNA samples from peripheral DCs

Blood samples were collected from each human subject through the ulnar vein puncture. Peripheral blood mononuclear cells (PBMCs) were isolated by the gradient density Percoll centrifugation. DCs were purified from the PBMCs by flow cytometry using CD11c as a surface marker. RNAs were extracted from DCs with the TRIzol reagents.

### RNA sequencing (RNAseq)

RNA samples were extracted from DCs and subjected to RNAseq analysis. The procedures of RNAseq were carried out by the professional staff of the biotech company (BGI, Shenzhen, China). The data were analyzed by the technical staff of the company. The results of differentially expressed genes (DEGs) are presented by volcano plots and heatmaps. The data of RNAseq were verified by conventional RT-qPCR.

### Western blotting

Proteins were extracted from cells collected from relevant experiments, separate by SDS-PAGE (sodium dodecyl sulfate–polyacrylamide gel electrophoresis), and transferred onto a PVDF (polyvinylidene difluoride) membrane. After blocking with 5% skim milk for 30 min, the membrane was incubated with the primary Abs (diluted to 200 ng/ml; the types of Abs are indicated in figures and legends) overnight, followed by incubating with HRP (horseradish peroxidase) conjugated second Abs (diluted to 20 ng/ml) for 2 h. Washing with TBST (Tris-buffered saline containing 0.05% Tween 20) three times was performed after each incubation. Enhanced chemiluminescence was utilized to develop the immunoblots on the membrane and were photographed using an imaging device (UVP, Cambridge, UK). Immunoblots were subjected to densitometry using ImageJ software when necessary.

### Flow cytometry (FCM)

Single cells were prepared from relevant experiments. In the surface staining, cells were incubated with Abs of interest (Ab types are detailed in figures and legends; diluted to 0.5 µg/ml) or isotype IgG for 30 min at 4 °C. The cells were washed with FCM buffer (phosphate buffered saline, PBS, containing 2% bovine serum albumin, BSA) three times, and analyzed with a flow cytometer (BD FACSCanto II). In the intracellular staining, cells were fixed with 1% paraformaldehyde (containing 0.05% Triton X-100 to increase the permeability of the membrane) for 1 h. The cells were then processed with the same procedures as the surface staining. The data were analyzed with Flowjo software (TreeStar Inc., Ashland, OR). The data obtained from the isotype IgG staining were used as the gating references.

### Mice

Male C57/BL6 mice (6–8 weeks old) were purchased from the Guangdong Experimental Animal Center (Foshan, China). *Tert*^f/f^ and *Itgax*-Cre mice were purchased from Jackson Laboratory (Bar Harbor, Main). A mouse strain carrying DCs deficient of *Tert* gene expression (the *Tert*^f/f^*Itgax*-Cre mice) was generated in-house by crossing *Tert*^f/f^ mice with *Itgax*-Cre mice. *Tert*^f/f^*Itgax*-Cre mice were used in experiments after 5 generations. The gene knocking out system was activated by feeding *Tert*^f/f^*Itgax*-Cre mice with tamoxifen (Sigma Aldrich; 20 mg/mouse in 0.3 ml corn oil) daily for 5 consecutive days. Mice were maintained in a specific pathogen-free facility at Shenzhen University with free access to food and water.

### Cell culture

The cells were cultured in a RPMI1640 medium supplemented by 2 mM of L-glutamine, 10% fetal calf serum, 0.1% streptomycin and 100 U/ml penicillin. Cell viability exceeded 99% based on the Trypan blue exclusion test.

### Enzyme-linked immunosorbent assay (ELISA)

The amounts of serum specific IgE (sIgE) and cytokines in the serum or culture supernatant were evaluated by ELISA using commercial reagent kits based on the recommended protocols.

### Immunoprecipitation (IP)

Proteins were extracted from cells harvested from relevant experiments and precleared by incubating with protein G agarose beads for 2 h. Supernatant was collected and incubated with Abs (1 µg/ml) of interest or isotype IgG overnight. The immune complexes were precipitated by incubating with protein G agarose beads for 2 h. The beads were collected by centrifugation at 5,000 *g* for 10 min. Proteins were eluted from the beads, and subjected to the analysis of Western blotting.

### Statistics

The difference between two groups was determined by the Student’s *t*-test, or Mann Whitney test. ANOVA followed by Dunnett’s test or Bonferroni test was performed for the data of more than two groups. Pearson correlation coefficient assay or Spearman correlation coefficient assay was conducted to determine the correlation between groups.

### Electronic supplementary material

Below is the link to the electronic supplementary material.

Supplementary Material 1

Supplementary Material 2

## Data Availability

No datasets were generated or analysed during the current study.
