# Supplementary figures and images for: An association between elevated telomerase reverse transcriptase expression and the immune tolerance disruption of dendritic cells
Source: Cell Commun Signal. 2024 May 23;22:284. doi: 10.1186/s12964-024-01650-6 (PMC11112790; doi:10.1186/s12964-024-01650-6)

fig1D

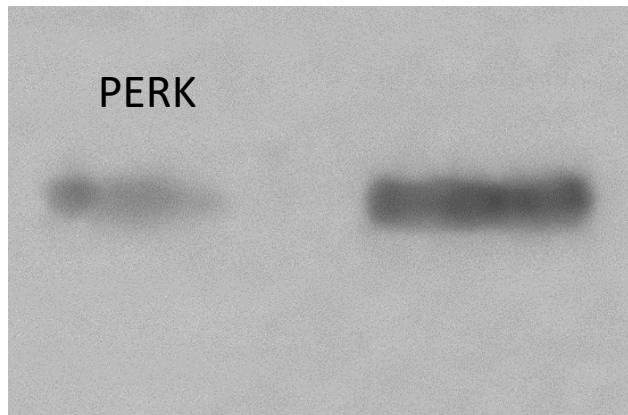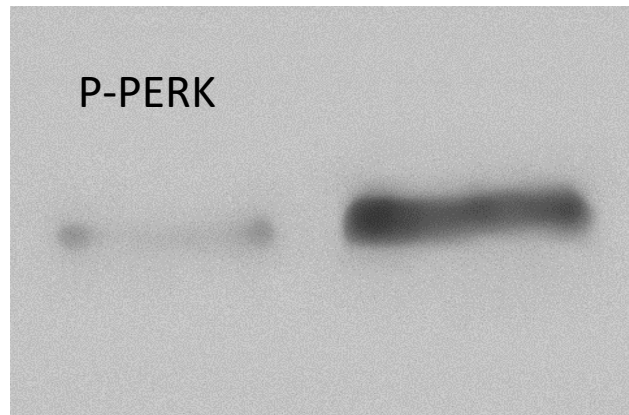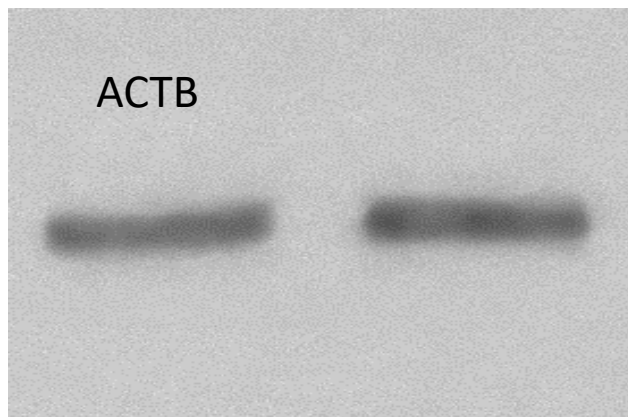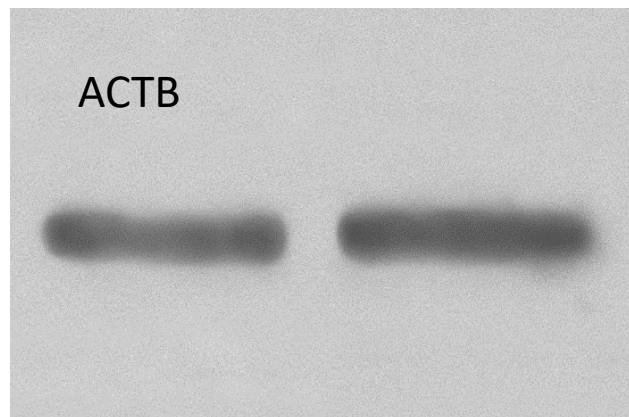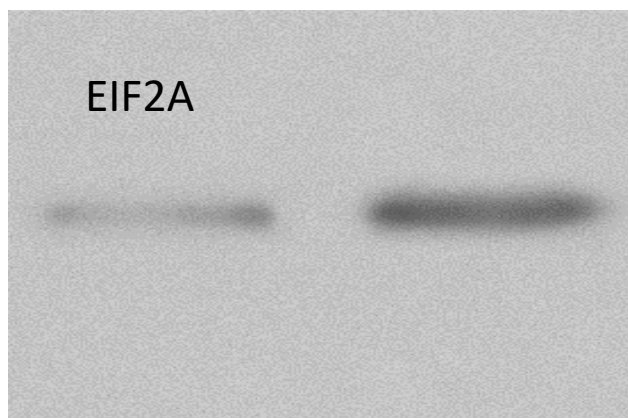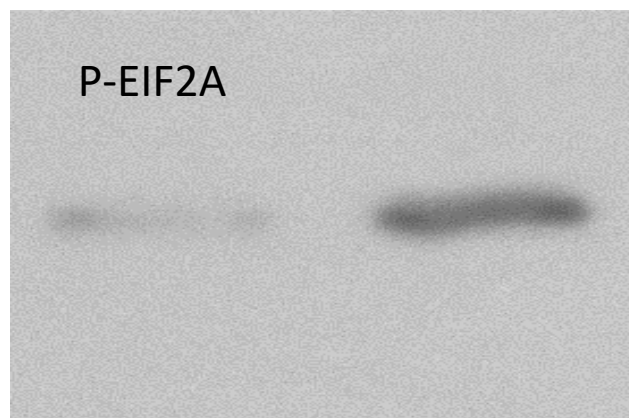

fig3

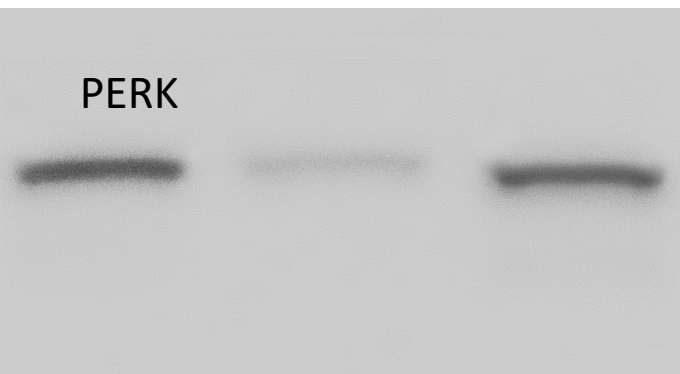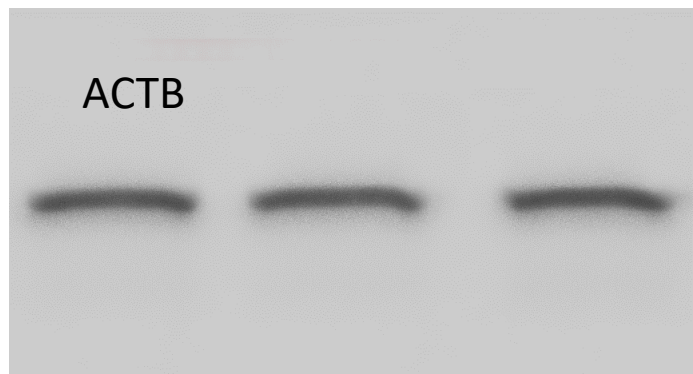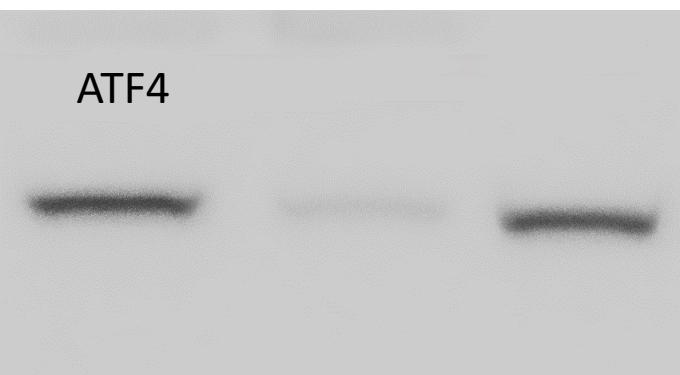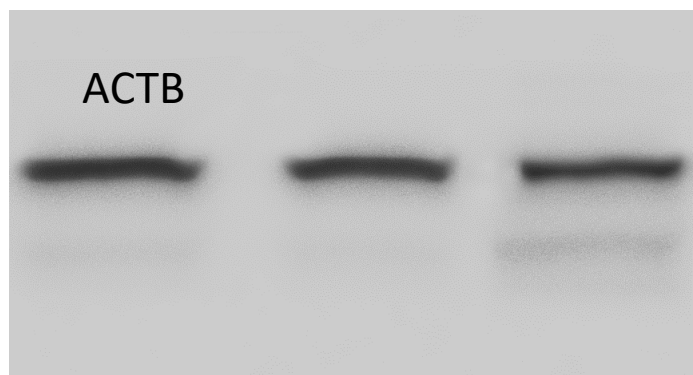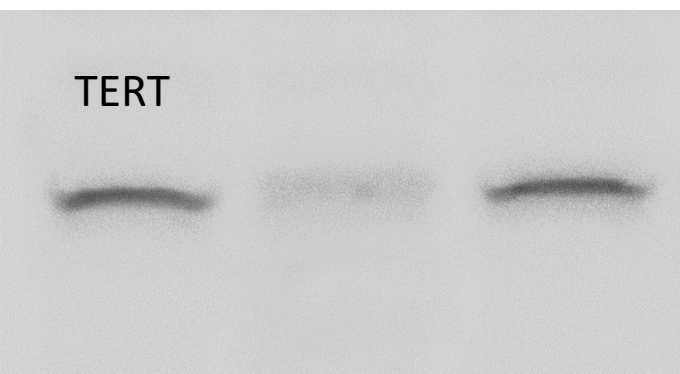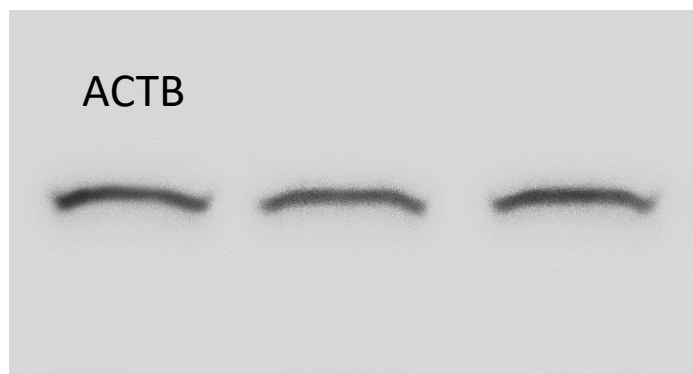

fig4

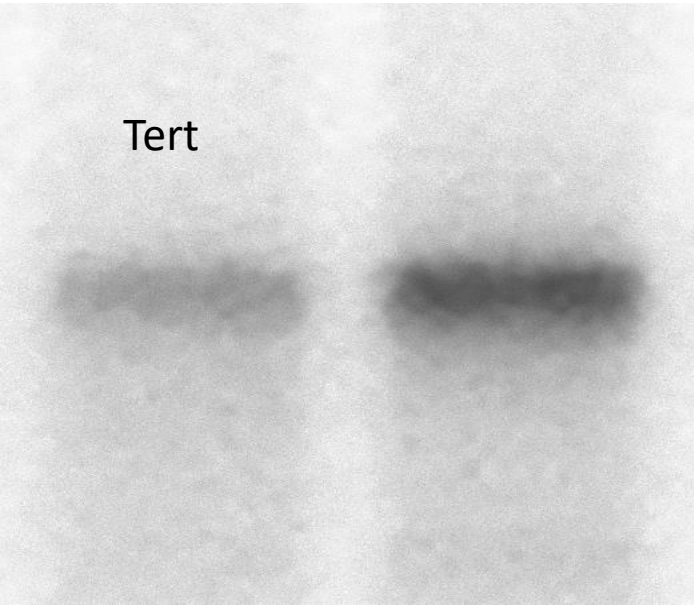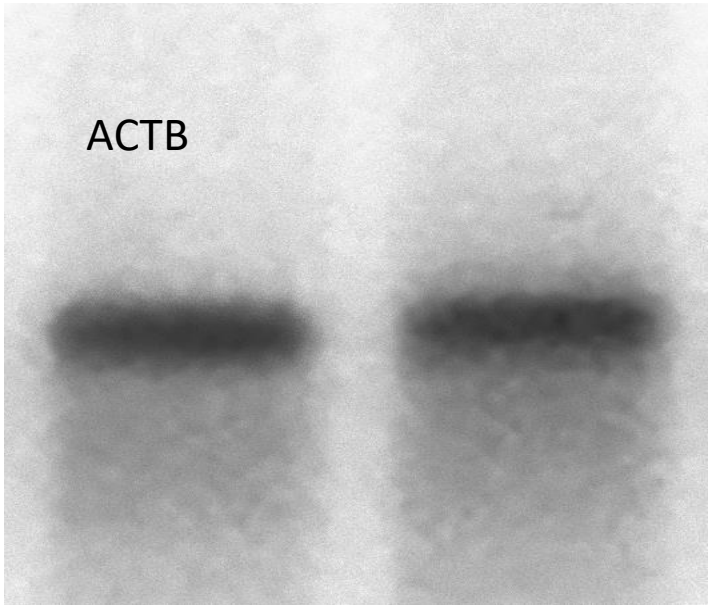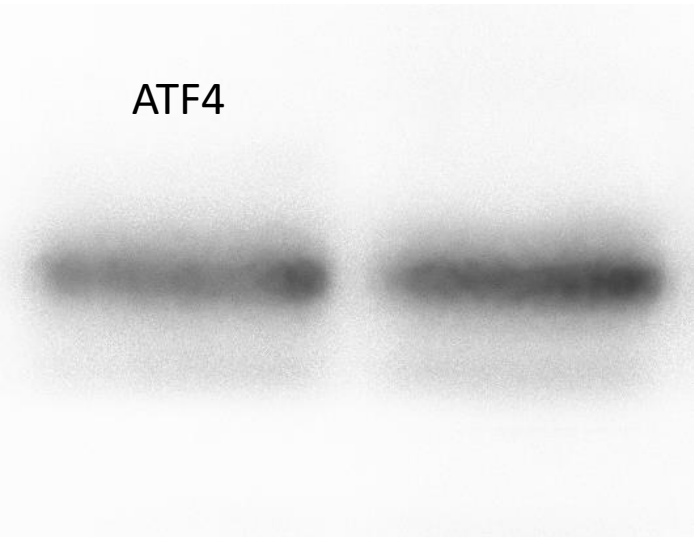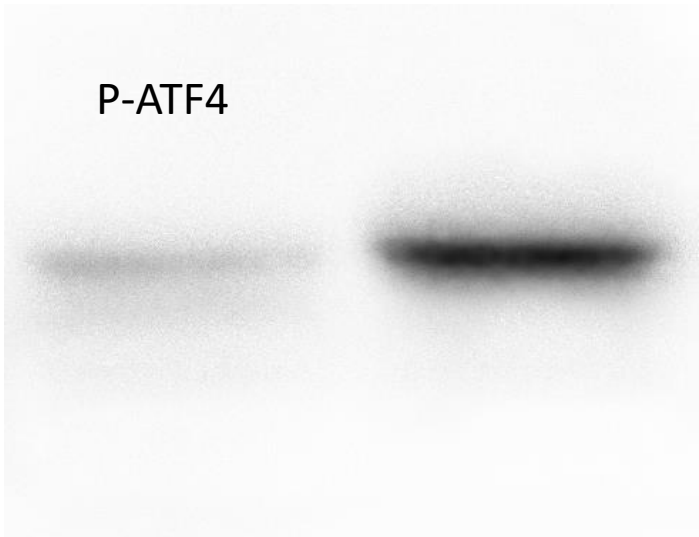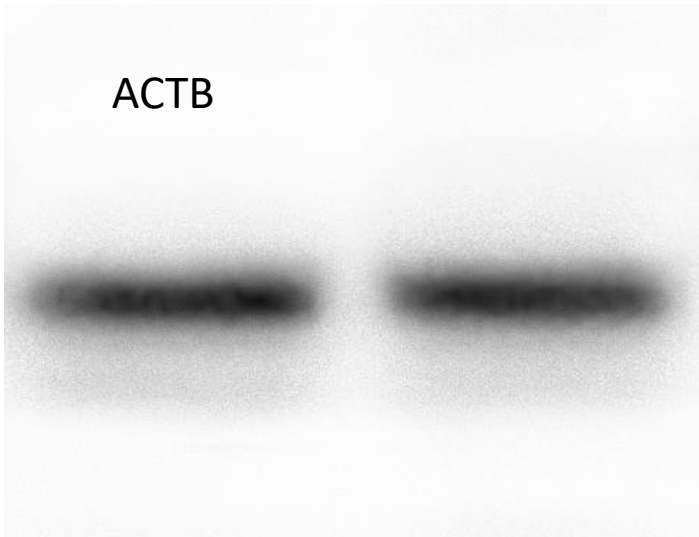

fig4

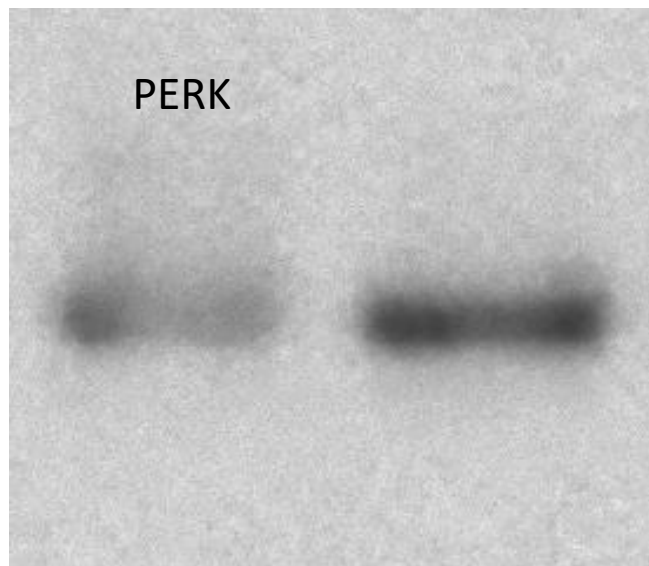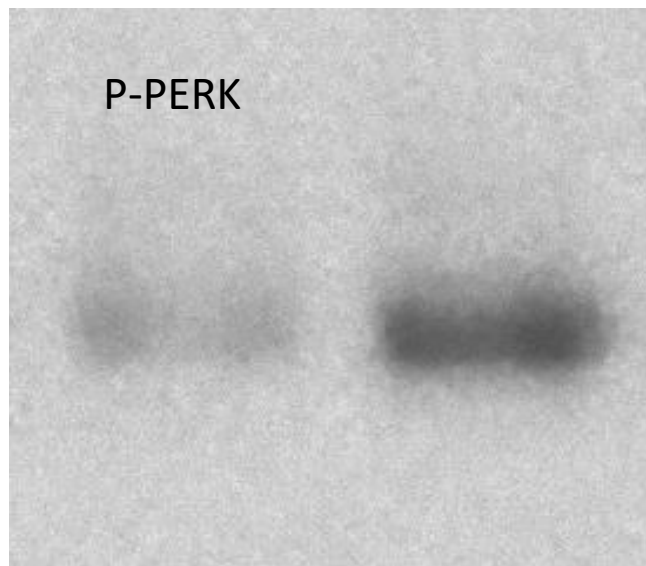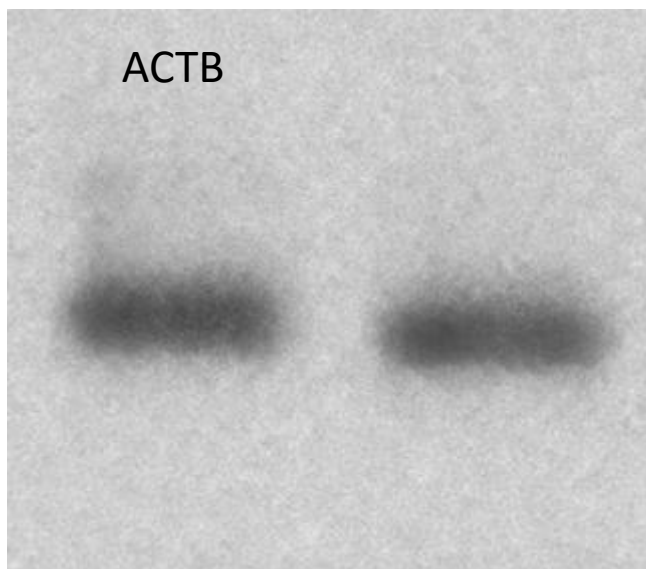

fig5A-B

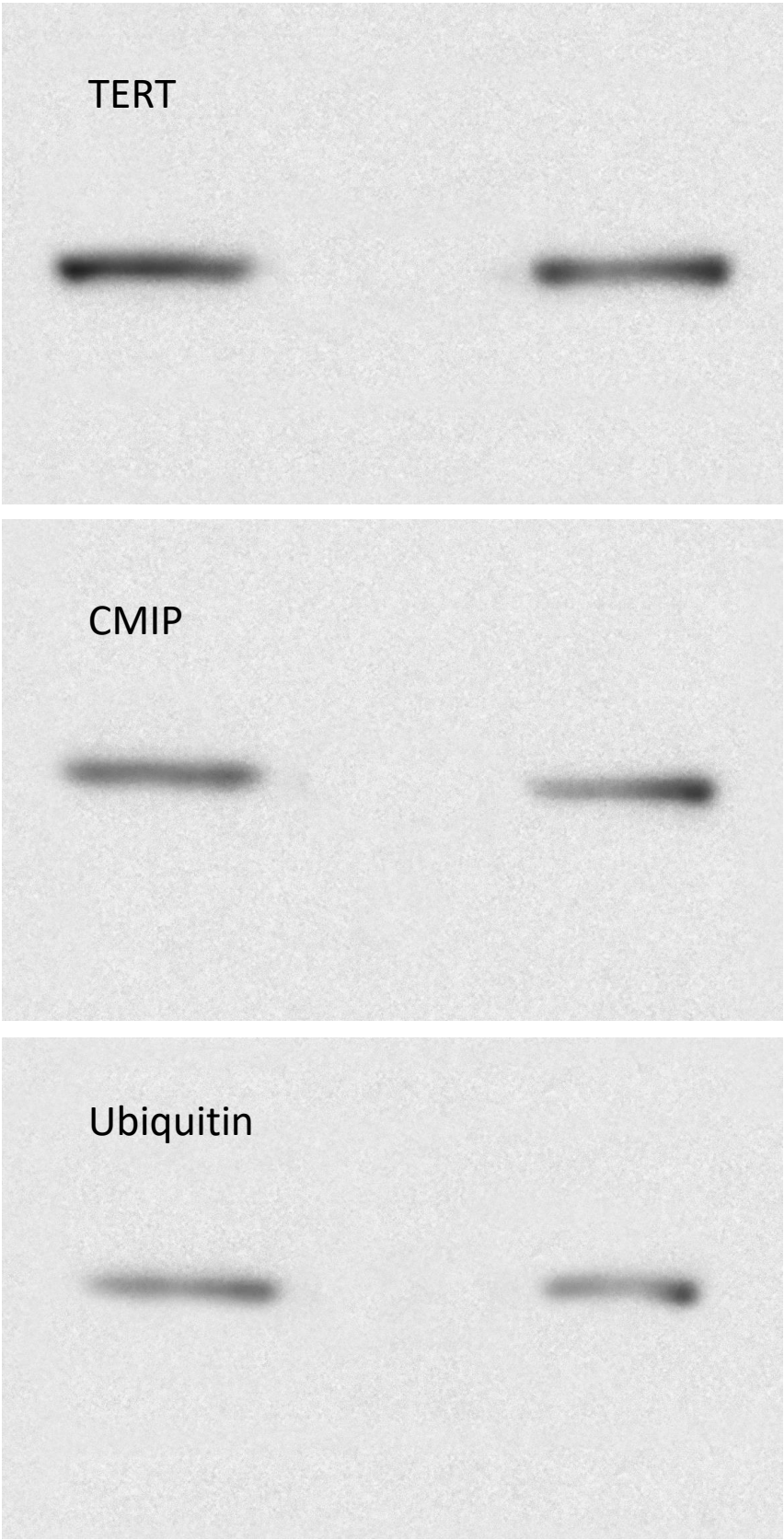

fig5C

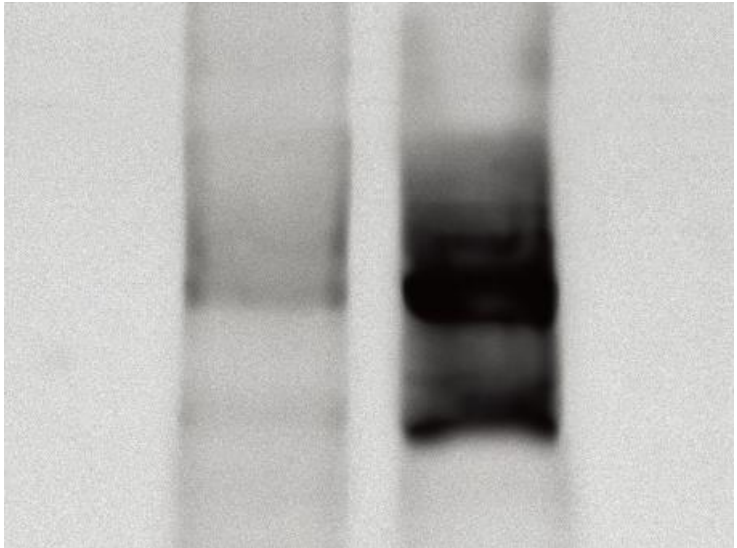

Ubiquitin

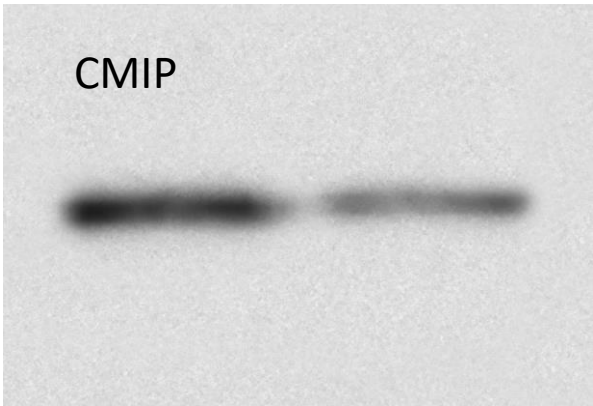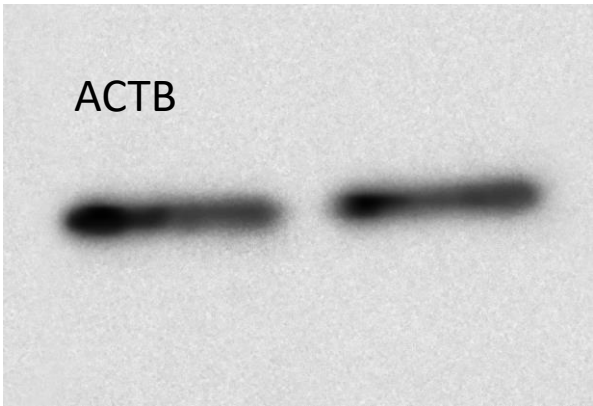

Supplement: Supplementary file 1 — Supplementary Material 1 [file 12964_2024_1650_MOESM1_ESM.pdf]
