# Supplementary material for: An association between elevated telomerase reverse transcriptase expression and the immune tolerance disruption of dendritic cells
Source: Cell Commun Signal. 2024 May 23;22:284. doi: 10.1186/s12964-024-01650-6 (PMC11112790; doi:10.1186/s12964-024-01650-6)
Supplement: Supplementary file 2 — Supplementary Material 2 [file 12964_2024_1650_MOESM2_ESM.docx]

**Supplemental materials**


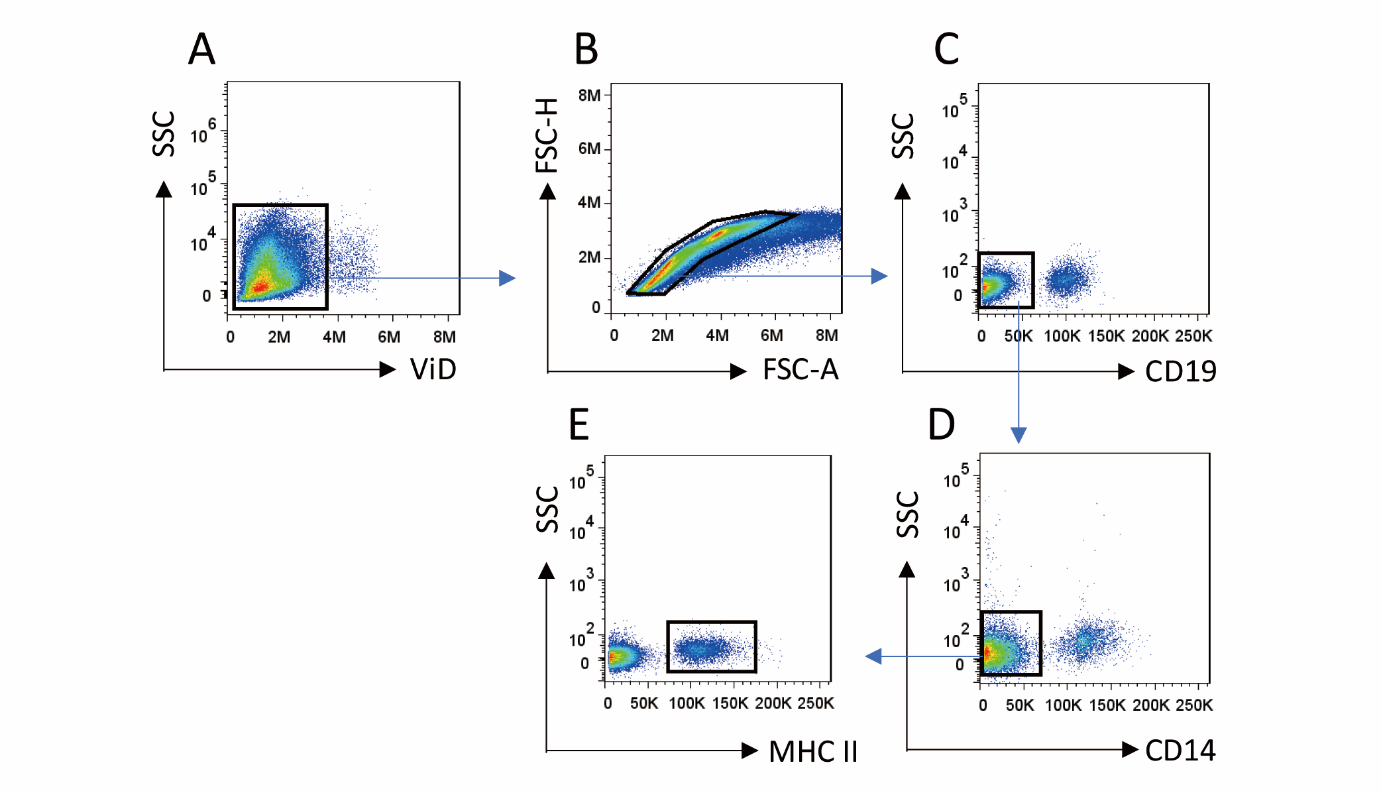


**Figure S1. Gating strategy of DCs**. PBMCs were prepared with blood samples, and stained with indicated Abs. Cells were processed by FCM cell sorting. A, live cells were gated. B, non-adherent cells were gated. C, non-CD19^+^ B cells were gated. D, non-CD14^+^ cells were gated. E, MHCII^+^ cells were gated, and sorted as dendritic cells.

Abbreviations: ViD: An amine reactive viability dye used to stain dead cells. DC: Dendritic cell. PBMC: Peripheral blood mononuclear cell. FCM: Flow cytometry.


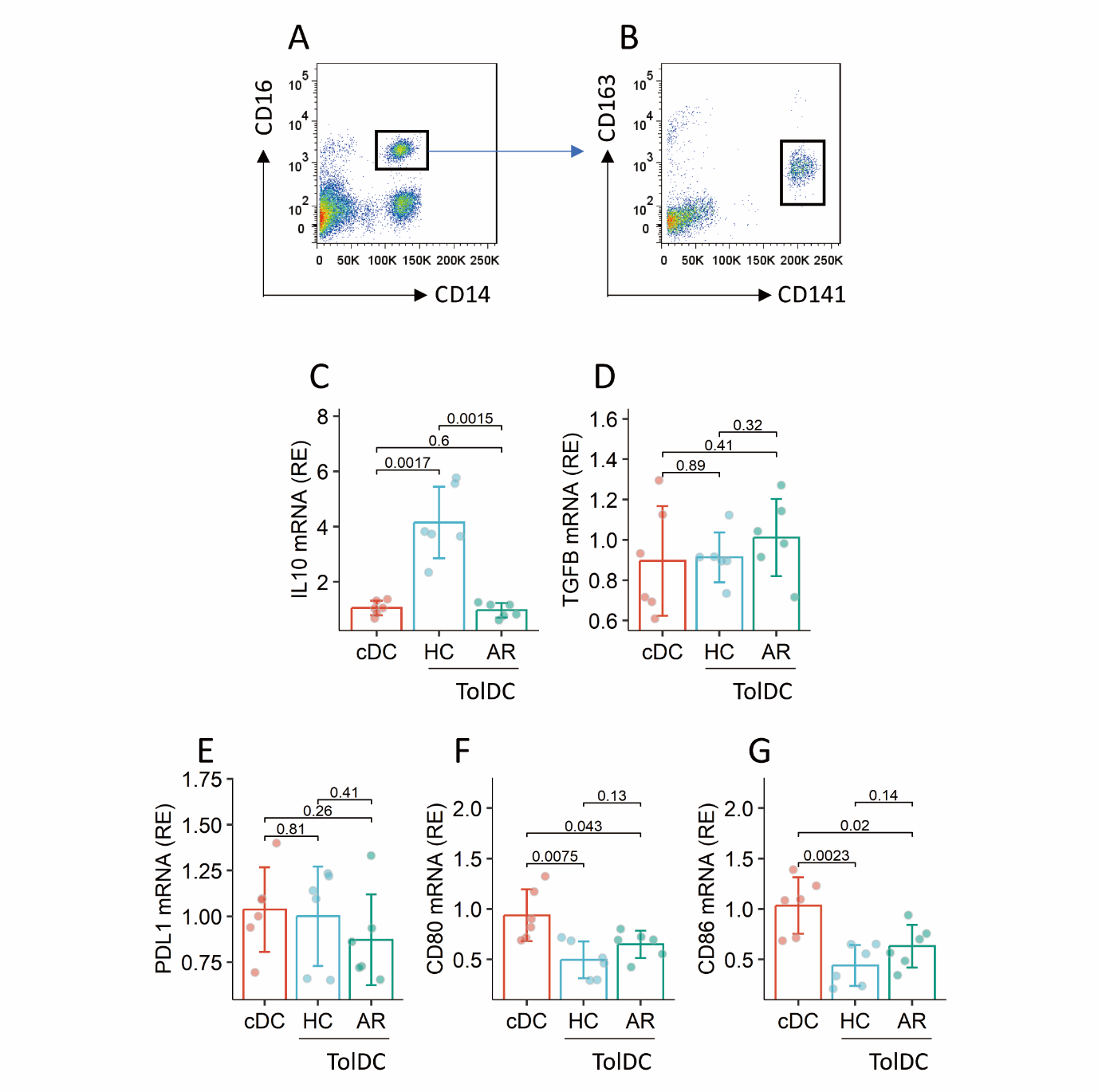


**Figure S2. Assessment of surface markers of TolDCs**. TolDCs (A; CD14^+^CD16^+^CD141^+^CD163^+^) and cDCs (CD11c^+^CD19־CD14־) were isolated from PBMCs. RNAs were extracted from the DCs, and analyzed by RT-qPCR. Bars show mean ± SD of mRNA amounts of indicated molecules. Each dot in bars presents one sample (Each sample was tested in triplicate). Statistics: Student’s *t*-test. p values are presented in figures where appropriate.

Abbreviations: cDC: Conventional DC. TolDC: Tolerogenic DC. PBMC: Peripheral blood mononuclear cell.


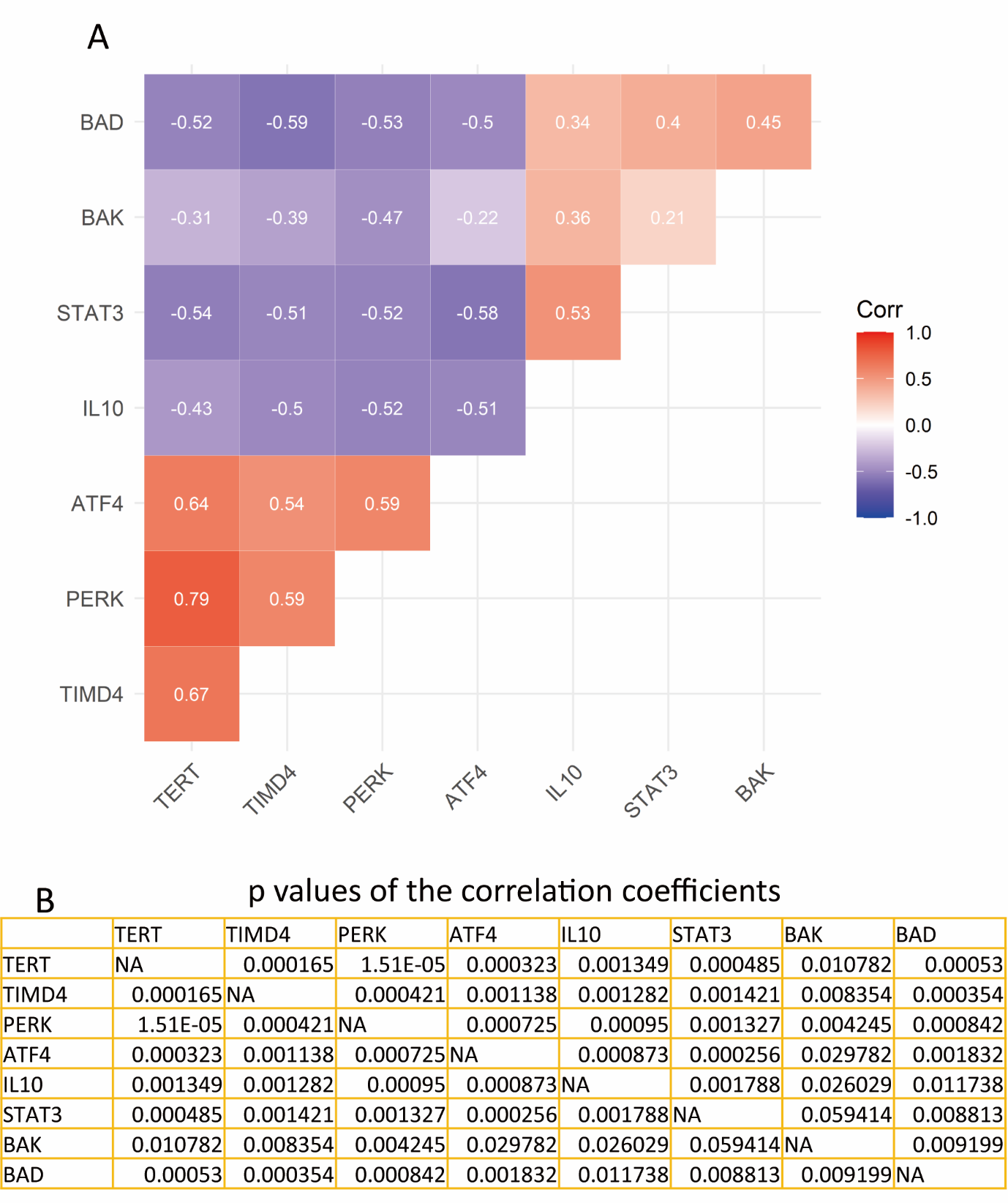


**Figure S3. Correlation between TERT and other 7 DEGs in DCs**. A correlation assay was performed with the mRNA values of DEGs in Fig. 1C. A, a heatmap shows correlation coefficients between DEGs. B, *p* values of the coefficients in A. Statistics: Spearman correlation coefficient test.


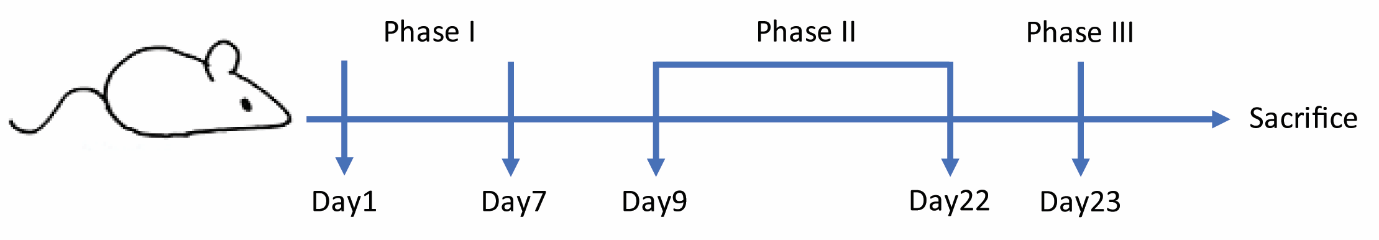


**Figure S4. A schematic of the OVA-alum protocol**. Phase I is the sensitization stage. Mice were subcutaneously injected with OVA (100 μg/mouse in 0.1 ml alum) on day 1 and day 7, respectively. Phase II is the immune boosting stage. Mice receive nasal instillations (20 μl/nostril that contain 5 mg OVA/ml) daily from day 9 to day 22. Phase III is the challenge stage. Mice receive nasal instillations (20 μl/nostril that contain 50 mg OVA/ml) on day 23, one time. The AR response was measured following the challenge, including counting nasal scratch times, sneezing like action within 30 min after the challenge. Mice were then anesthetized by peritoneal injection with ketamine (100 mg/kg). Blood samples were collected with the eyeball-pulling procedure. Nasal lavage fluids were collected afterwards. Mice were then sacrificed by cervical dislocation.


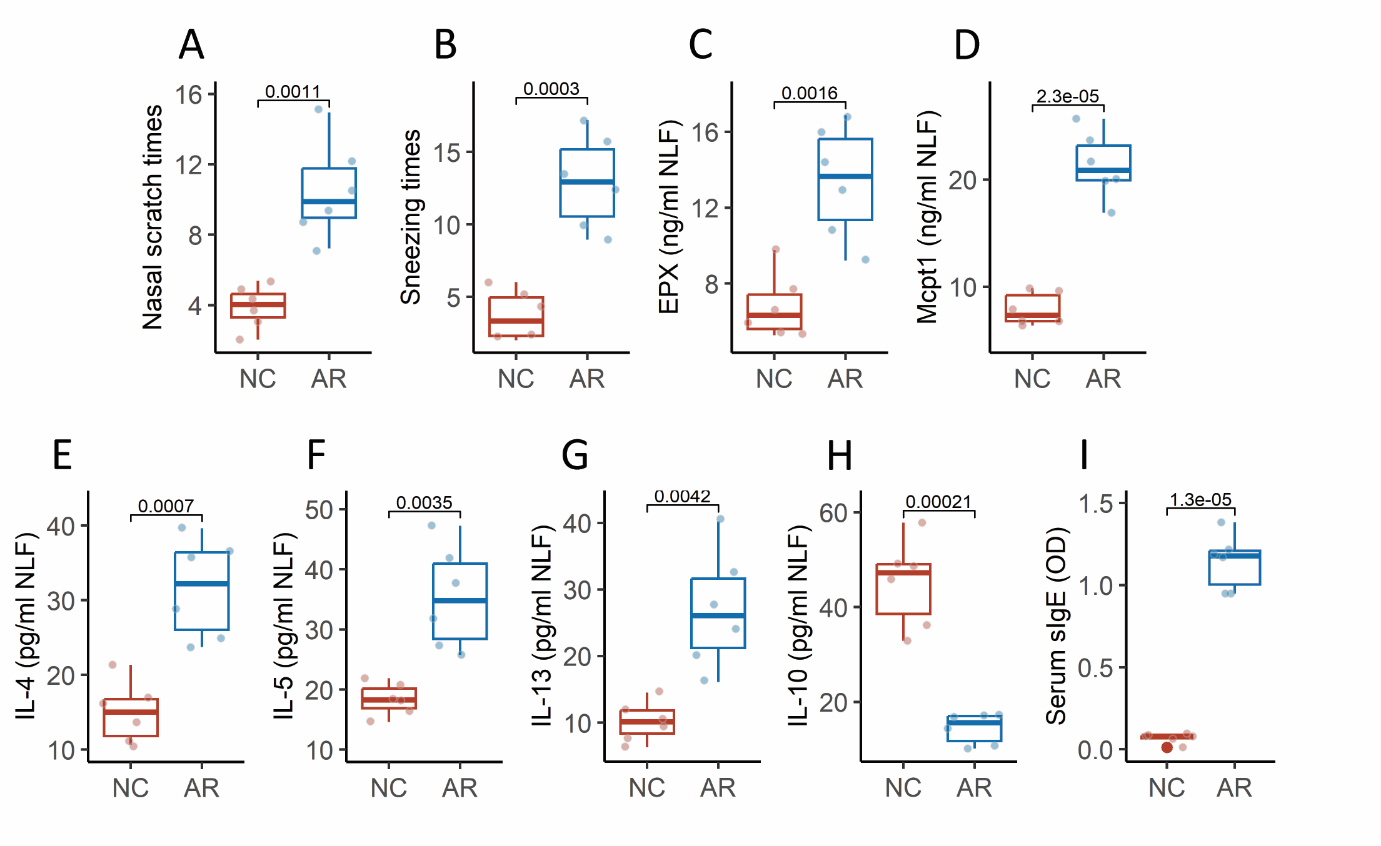


**Figure S5. Establishment of an AR mouse model**. An AR mouse model was established with the OVA-alum protocol (Fig. S4). Boxplots show AR associated molecule amounts, including the AR like symptoms (nasal itch and sneezing; A and B), allergic mediators (EPX and Mcpt1, C and D), Th2 cytokines (IL-4, IL-5, and IL-13, E-G), and the immune suppressive molecule IL-10, in NLF, and the quantity of sIgE (H). The data of boxplots are presented as median (IQR) of 6 mice per group. Statistics: Student *t*-test. *P* values are presented in figures where appropriate. Each dot in boxplots presents one sample (Each sample was tested in triplicate).

Abbreviations: NC: Normal control. AR: Allergic rhinitis. OVA: Ovalbumin. alum: Aluminum hydroxide. NLF: Nasal lavage fluid. sIgE: Specific IgE.


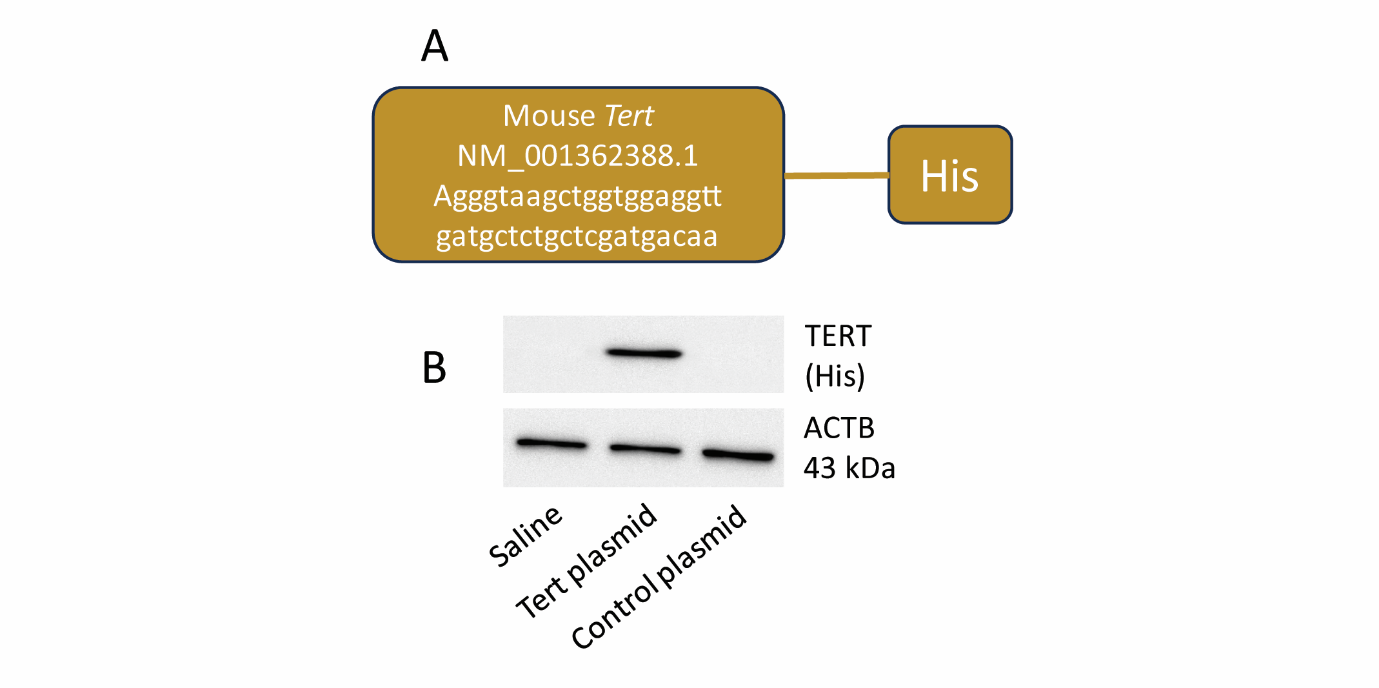


**Figure S6. Enforced production of TERT in DCs in vitro**. A, the *Tert*-expressing plasmid information. B, immunoblots show recombinant TERT in DCs.


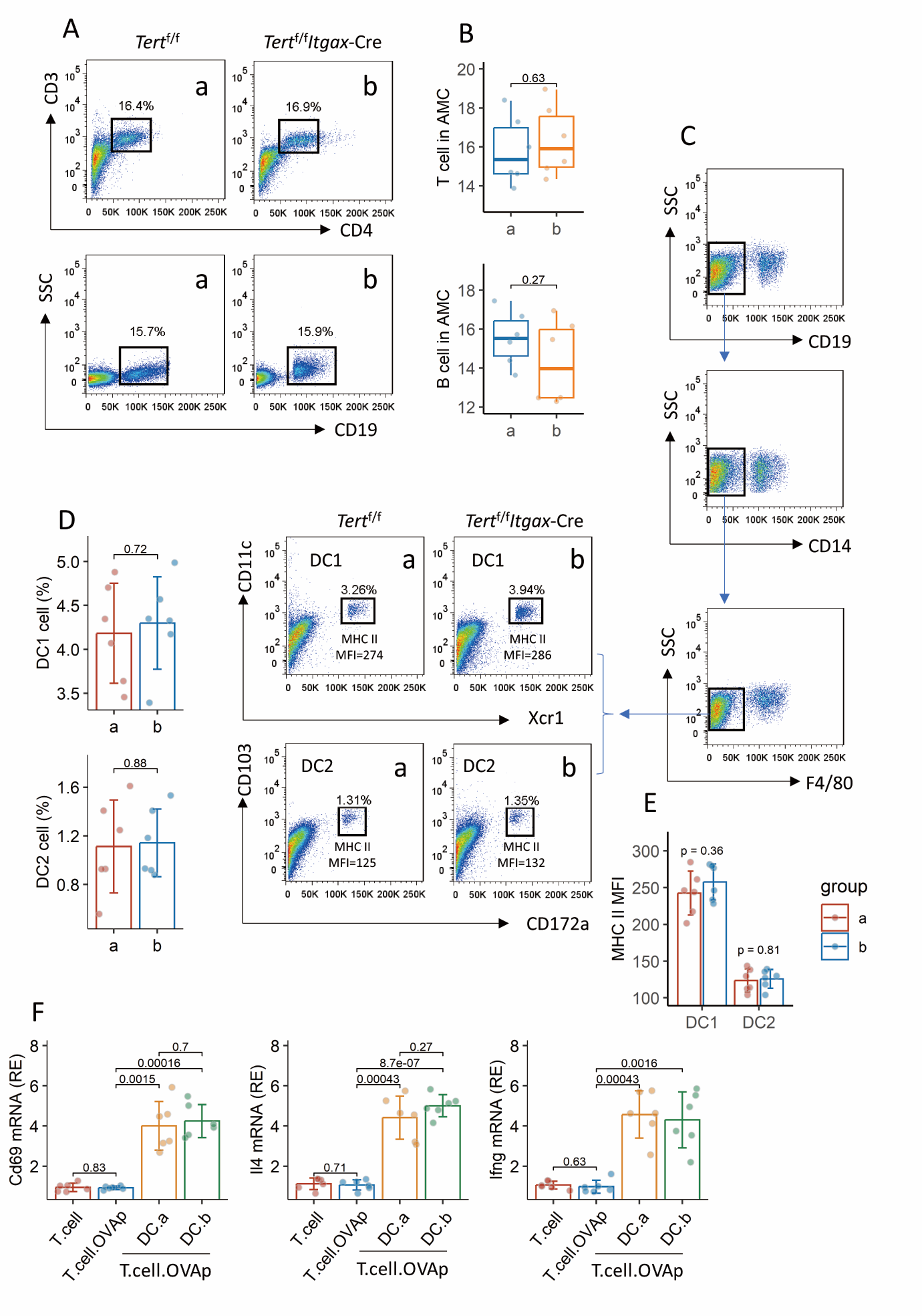


**Figure S7. Assessment of immune cells in AMCs of *Tert*^f/f^ mice and *Tert*^f/f^ *Itgax*-Cre mice**. AMCs were prepared with the airway tissues of *Tert*^f/f^ mice and *Tert*^f/f^ *Itgax*-Cre mice, and analyzed by FCM. A, gated FCM plots show CD4^+^ T cell and B cell. B, boxplots show median (IQR) of CD4^+^ T cell and B cell counts from 6 mice per group. C, gating strategy of DCs, and DC1 and DC2 plots. D, median (IQR) of DC1/DC2 counts of 6 mice per group. E, MHC II MFI of DC1/DC2. F, CD4^+^ T cells were isolated from OT-II mouse spleen, and cultured with DCs prepared from *Tert*^f/f^ mice or *Tert*^f/f^ *Itgax*-Cre mice in the presence of OVA peptide overnight. CD4^+^ T cells were then isolated and analyzed by RT-qPCR. Bars indicate the mRNA levels of *Cd69*, *Il4* and *Ifng*, respectively. Statistics: Student *t*-test. *p* values are presented in figures where appropriate. Each dot in plots presents one sample (Each sample was tested in triplicate).

Abbreviations: AMC: Airway mononuclear cell. FCM: Flow cytometry. DC: Dendritic cell. MFI: Median fluorescence intensity.


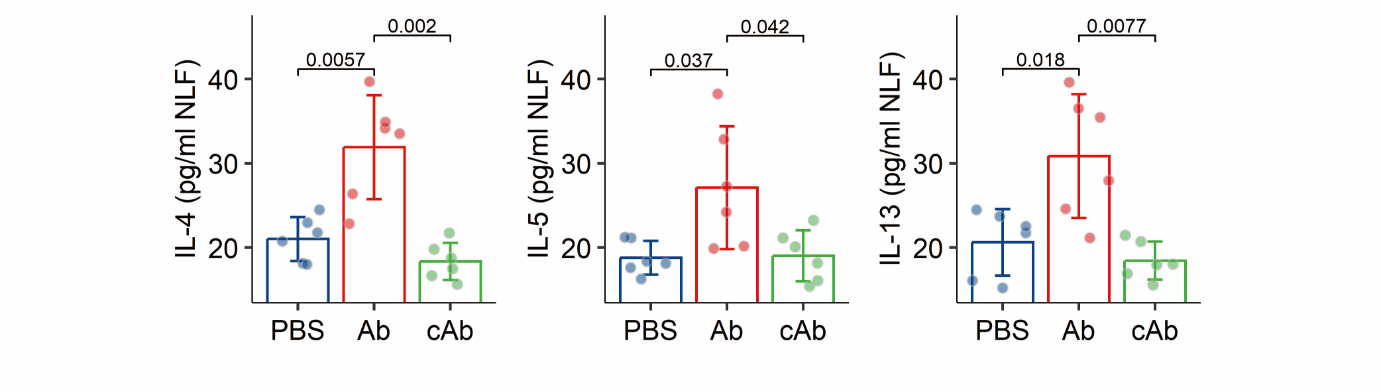


**Figure S8. Inhibition of IL-10 induces Th2 bias in the airways**. Naïve mice (n=6) were treated with nasal instillations (20 µl/nostril; containing a neutralizing anti-IL-10 Ab, JES5-2A5, 100 µg/ml) daily for 7 consecutive days. NLF was collected from each mouse immediately after the sacrifice, and analyzed by ELISA. Bars show mean ± SD of the amounts of indicated cytokines from 6 mice per group. Each dot in bars presents one sample (Each sample was tested in triplicate). Statistics: ANOVA + Bonferroni test. p values are presented in figures where appropriate.

Abbreviations: Ab: the JES5-2A5 neutralizing anti-IL-10 antibody. cAb: An isotype IgG; used as a control Ab. NLF: Nasal lavage fluid.

Table S1. Primers used in the present study

| Molecules | Forward | Reverse |
| --- | --- | --- |
| PERK | tctgttcagctctgggttgt | ccgaagttcaaagtggccaa |
| ATF4 | gtccctccaacaacagcaag | actttctgggagatggccaa |
| TERT | cgtggtttctgtgtggtgtc | ccttgtcgcctgaggagtag |
| TIMD4 | cctttcactaaccccaagca | ccaggctgaggagaagacac |
| IL10 | tgccttcagcagagtgaaga | ggtcttggttctcagcttgg |
| CMIP | ccagtttgcttcaacccatt | gtaacaggagcccatgagga |
| BAK | ttttccgcagctacgttttt | ggtggcaatcttggtgaagt |
| BAD | ccgagtgagcaggaagactc | ggtaggagctgtggcgact |
| TGFB | gggactatccacctgcaaga | cctccttggcgtagtagtcg |
| PDL1 | cgaagtcatctggacaagca | atttggaggatgtgccagag |
| CD80 | agggaacatcaccatccaag | tgccagtagatgcgagtttg |
| CD86 | gtattttggcaggaccagga | attcctgtgggctttttgtg |
| STAT3 | tttcacttgggtggagaagg | gctacctgggtcagcttcag |
| ACTB | ggacttcgagcaagagatgg | agcactgtgttggcgtacag |
| Perk | cggagacagtgtttggctta | gctttttcccatcattctcg |
| Atf4 | tcgatgctctgtttcgaatg | ggcaacctggtcgactttta |
| Tert | agggtaagctggtggaggtt | gatgctctgctcgatgacaa |
| Il10 | ccaagccttatcggaaatga | ttttcacaggggagaaatcg |
| Cd69 | gtacaattgcccaggcttgt | tccaatgttccagttcacca |
| Il4 | tcaacccccagctagttgtc | tgttcttcgttgctgtgagg |
| Ifng | actggcaaaaggatggtgac | tgagctcattgaatgcttgg |
